# Modeling audio dynamics using hierarchical assisted K-means model for structured speaker profiling in TED talks

**DOI:** 10.1038/s41598-026-47033-4

**Published:** 2026-06-16

**Authors:** M. N. Renukadevi, T. M. Rajesh, S. G. Shaila, Praveen Kulkarni, Tina Babu, Rekha R. Nair, Sumendra Yogarayan

**Affiliations:** 1 Department of Computer science and Engineering, Dayananda Sagar University, Bangalore South, 560112 India; 2 Department of Computer science and Medical Engineering, Dayananda Sagar University, Bangalore South, 560112 India; 3 Department of Data Science and Engineering, Dayananda Sagar University, Bangalore South, 560112 India; 4 Alliance School of Advanced Computing, Bengaluru, India; 5 Centre for Intelligent Cloud Computing, COE for Advanced Cloud, Multimedia University, Bukit Beruang, Melaka, Malaysia

**Keywords:** Zero Crossing Rate (ZCR), Short-Term Energy (STE), Hierarchical-K-means clustering, Two-Stage Hybrid Clustering Architecture, Speaker Profiling, Vocal traits, TED Talks, Engineering, Mathematics and computing

## Abstract

Speaker vocal delivery significantly impacts audience engagement in knowledge-sharing platforms like TED Talks. However, existing classification approaches face limitations in noise handling, feature extraction, and clustering robustness. The study investigates vocal behavior using a curated set of 5,000 TED Talk audio clips from 500 diverse speakers, analyzing voiced-unvoiced segments based on acoustic features including Zero Crossing Rate (ZCR), Short-Term Energy (STE), spectral measures, and auto correlation-based periodicity. Consistent patterns were observed across all speakers: voiced segments exhibited higher energy and strong periodicity, whereas unvoiced frames showed elevated zero-crossing activity and wide spectral spread. These features served as input for extensive clustering experiments aimed at profiling speaker vocal characteristics. The Proposed research aimed to address these gaps by developing a robust and interpretable hybrid clustering framework that combines K-means initialization (k = 6) with hierarchical agglomerative refinement, ultimately profiling TED speakers into six interpretable vocal types: Energetic, Balanced, Rhythmic, Flat, Noisy, and Muffled. Four baseline clustering methods (K-means, Spectral, Agglomerative, DBSCAN) demonstrated only moderate performance with silhouette scores of 0.52-0.60. In contrast, the proposed hybrid method achieved markedly superior results, a silhouette score of 0.90 ± 0.02, Davies -Bouldin (DB) index of 0.92 ± 0.04, and Calinski-Harabasz (CH) score of 1,020.5 ± 25.0. Feature-ablation tests further validated the importance of Mean Power and Magnitude, as removing both features degraded clustering quality by 5.6% and increased the DB index by 30.4%, confirming that the full-feature Hybrid approach delivers the most robust structure.

## Introduction

Speaker classification and speech analysis enhance content accessibility and allow personalized recommendations in the context of TED Talks. Speech analysis serves as a fundamental component in various applications, including speech emotion recognition, speaker profiling, and personalized content recommendation systems. Recommendation systems can suggest talks to users by profiling speakers through speech characteristics like tone, pace, and emotional expression. Accurate speaker classification improves user experience and increases interaction quality among various audiences.

Vocal delivery has been acknowledged as one of the main elements of successful communication, however, present methods lacks systematic quantifiable frameworks. Experienced listeners can usually distinguish effective delivery from an ineffective delivery, but they often cannot explain the specific acoustic characteristics that differentiate them. This limitation makes it difficult to provide actionable feedback to novice speakers. This gap motivates the development of computational methods which not only classify speaker vocal patterns but also create interpretable profiles for targeted improvement. This work aims to bridge the gap between expert intuition and systematic speaker development by creating an objective framework of vocal types based on measurable acoustic features^1^.

Despite ongoing research in this domain, existing methods face several critical obstacles in speaker profiling. Most studies rely on homogeneous datasets, which limit data diversity and the ability to adapt to different speaking styles, speech lengths, and recording conditions. Furthermore, feature extraction techniques are often incomplete and fail to identify the important vocal traits such as spectral and temporal characteristics necessary for accurate classification. Mel-Frequency Cepstral Coefficients (MFCC) and Linear Predictive Coding (LPC) which are frequently used, may overlook important audio features such as power spectral density and magnitude, which are essential for precise acoustic profiling. Data quality is often compromised, making it difficult to identify the most relevant the speech patterns, especially in a noisy environment. Basic pre-processing techniques fail to effectively separate voiced from unvoiced signals, however, this separation is essential for effective feature extraction and recognition accuracy. Moreover, excessive reliance on simple clustering method prevents the discovery of the complex speech, resulting in less precise and difficult-to-interpret classification outcomes. Traditional clustering methods often fail to capture the non-linear structure of speech data and are limited in their modeling ability.

Additionally, while existing clustering methods (e.g., K-means, spectral clustering, agglomerative hierarchical clustering) have been utilized for speaker segmentation, they typically have a several limitations: they depend on a limited set of features (e.g., only MFCCs or speaker embeddings) instead of comprehensive acoustic-temporal representations, lack interpretability in terms of how the clusters correspond to speaker vocal styles, and there are no systematic benchmarks for clustering quality and comparative evaluation in the speaker profiling domain. Hence, three primary research gaps were identified as follows: (1) current studies heavily depend on limited and homogeneous audio features and do not adequately capture the full spectral-temporal characteristics for speaker profiling; (2) present solutions do not have strong noise reduction and voiced-unvoiced separation capabilities, which results in the low-quality feature extraction; (3) conventional clustering techniques are less interpretable, lack comprehensive feature representation, and are not rigorously benchmarked for the speaker style categorization.

Traditional clustering methods have been employed in speaker analysis but are generally constrained either by their computational scalability or by their capability to capture hierarchical vocal patterns. Standard K-means clustering, while being efficient, struggles to identify nested structures within speaker groups. Conversely, pure hierarchical methods encounter computational problems when handling large datasets and might result in overly fragmented clusters. Hybrid clustering innovations have been successful in other areas, but few studies have investigated their use in acoustic speaker profiling.

In order to overcome these problems, the present study develops a unified framework for the description and classification of speaker vocal features based on advanced acoustic feature extraction and a novel hybrid clustering approach specifically designed for TED speakers. This work utilizes well-chosen set of audio features. Zero Crossing Rate (ZCR), Short-Term Energy (STE), Power Spectral Density, and different statistical measures such as mean, standard deviation, power, and magnitude identify the fundamental acoustic components that define speaker characteristics. The use of TED Talks as the data source is particularly beneficial, as it offers a rich and diverse dataset that reflects the variations in speaker background, presentation style, content tone, and audience engagement levels.

The proposed study addresses the gaps found in the research literature by the following three major innovations: First, it applies thorough preprocessing steps such as noise reduction and voiced-unvoiced separation to provide high-quality feature extraction from different acoustic environments^2^. Second, it derives a detailed 12-dimensional feature set representing both spectral and temporal characteristics, which are very important for complex speaker profiling. Third, it creates a novel two-stage hybrid clustering framework idea that merges the fast computation of K-means with the structural, awareness of hierarchical clustering. In contrast to the conventional bisecting K-means methods that require uniform binary splits at each level, the proposed methodology permits adaptive, non-uniform subdivision directed by acoustic homogeneity criteria. Initially, this hybrid framework uses K-means clustering (K=6) followed by independent hierarchical refinement within each macro-cluster. Unlike bisecting K-means (uniform binary splits) or global hierarchical methods (single dendrogram for all speakers), in the proposed methodology, each macro-cluster has its own individual dendrogram, using adaptive discontinuous cutting, to evaluate each macro-cluster using m = 1 to 6 subclusters. Each macro-cluster is evaluated independently to identify the m* that maximizes the local silhouette score for sub clustering; therefore, the maximum number of subclusters per macro-cluster is 6, although no upper limit is imposed globally. There are three reasons for the choice of 6 as the upper limit of subclusters per macro-cluster. i) The first is based on empirical evidence; in experiments with the 500-speaker dataset, the improvement in silhouette score for the dataset consistently levelled out beyond m = 6 subclusters. ii) The second reason is related to the interpretability of clusters; when there are more than 6 subclusters per macro-cluster, the resulting categories begin to show duplication in terms of their acoustic characteristics, making it difficult to distinguish between different types of speakers. iii) The third reason is related to the need for the computational efficiency of the number of speakers in the dataset, which is large. If the marginal improvement in the silhouette score drops below 0.01 between two successive cuts, the sub clustering will stop and a smaller m* will be used, thus ensuring that any macro-cluster that is homogeneous remains so (m* = 1), while heterogeneous macro-clusters will only be subdivided as necessary. This leads to non-uniform sub clustering, whereby the homogeneous clusters will remain unchanged while the heterogeneous clusters will be divided into meaningful subclusters. This yields six interpretable vocal types: Energetic, Balanced, Rhythmic, Flat, Noisy, and Muffled, have been obtained.

### Novel contributions

The study makes four key contributions to vocal delivery profiling.

i) A novel two- stage Hybrid Hierarchical-K-means (HC-KM) framework that innovatively merges K-means macro-clustering (k = 6) with hierarchical refinement, independently for per macro-cluster. This method allows adaptive non-uniform subdivision (1–6 subclusters per cluster) based on local silhouette optimization rather than simple binary splits, resulting in 1.54 computational efficiency of the full hierarchical method. The upper bound of 6 is a per cluster maximum empirically determined by silhouette score saturation beyond m=6 in preliminary experiments, with early stopping applied when marginal silhouette gain falls below 0.01. This results in 1.54$\times$ computational efficiency over the full hierarchical method
ii) Comprehensive Acoustic Feature Set: (ZCR, STE, power spectral density, mean power, magnitude, standard deviation) valid through ablation;
iii) Superior Clustering Performance with Statistical Validation: Obtained silhouette score 0.90±0.02, Davies-Bouldin index 0.92±0.04, and Calinski-Harabasz score 1020.5±25.0, confirmed by 30 bootstrap runs with 95% confidence intervals.
iv) six interpretable vocal archetypes: (Energetic, Balanced, Rhythmic, Flat, Noisy, Muffled) where PCA demonstrated 80.2% variance captured in two dimensions, providing actionable feedback for communication training and speaker coaching.

The paper is organized as follows. Section 2 explores related work; Section 3 presents Methodology; Section 4 discusses results; Section 5 concludes with limitations and future directions.

## Related work

Speaker profiling and vocal delivery analysis have been investigated in various fields, encompassing emotion recognition, diarization, and speech enhancement. However, current approaches face three critical limitations. 1) reliance on narrow features sets (primarily MFCCs or speaker embeddings) that fail to capture comprehensive spectral-temporal characteristics. 2) Insufficient noise handling and voiced-unvoiced separation capabilities, and 3) lack of interpretable clustering frameworks with rigorous benchmarking for vocal style categorization.

### Feature extraction for speech analysis

Traditional speech analysis heavily relies on MFCCs and LPCs for acoustic characterization. Yadav et al. (2023)^3^ surveyed feature extraction techniques across speech recognition systems but noted limited integration of power-based features. Liu et al. (2024) used language model (LLM) based features (F0 and MFCC) for speech importance estimation but noted limited robustness across diverse inputs^4^. Their feature set lacked spectral-temporal descriptions essential for comprehensive vocal profiling. Recent work has emphasized complementary features: Ahmed & Lawaye (2023) demonstrated short-term energy effectiveness of endpoint detection^5,6^. While Chakravarty & Dua (2024) combined spectral and temporal features for deepfake detection^7^. However, comprehensive feature sets incorporating ZCR, STE, PSD, mean power, magnitude, and standard deviation for vocal style profiling remain unexplored, particularly in presentation contexts.

### Clustering approaches in speaker analysis

Speaker diarization and segmentation have employed various clustering strategies. Singh and Ganapathy (2021) proposed hierarchical clustering methods for speaker diarization but experienced computational scalability restrictions due to the O(n3) complexity^8^. Khadar et al. (2025)^9^ used agglomerative hierarchical clustering on TDNN x-vectors achieving moderate performance in noisy environments but lacking interpretability dimensions of vocal styles. Jiang et al.suggested using GMM(Gaussian Mixture-Model) based clustering to separate teacher-student voices, but the challenges of GMMs regarding dealing with many-to-many transitions and constructing complex acoustic manifolds limit their effectiveness compared with contemporary clustering techniques^10^. In 2024, Khalid et al. developed a fuzzy C-means clustering approach for video summarization and found improvements in cluster quality, however they did not have the capability to conduct hierarchical refinement operations^11^. Newer ideas such as those presented by Dhulipala et al. that present optimized dendrogram computational algorithms have the potential for more efficient organization of data compared with previous methods, yet no prior work has been conducted that systematically combines the speed of K-means clustering with the hierarchical flexibility of a Hierarchical cluster framework through the generation of adaptive subdivisions for speaker-specific vocal profiling^12,13^.

### TED talk analysis and vocal delivery

Studies on TED Talks have primarily focused on rhetorical strategies rather than acoustic profiling. Boyle et al (2024) manually annotated persuasive techniques achieving only moderate inter-annotator reliability (without an analysis of voice), without acoustic analysis. Nadeem (2023) conducted a qualitative analysis of narratives used during TED Talks around mental health, but he did not consider voice metrics in his analysis. Degano et al (2024) also used MEG to study the interaction of prosody and syntax^14–16^. However, this study only examined English native speakers and did not include data collected from other languages. Naderifarjad & Niknia (2024) conducted an assessment of the effects of watching TED videos for developing speaking abilities among EFL learners^17^. This study was conducted using a quasi-experimental design, but there was no acoustic delivery pattern analysis performed. No existing work has assessed TED Talk speakers into interpretable vocal categories based on comprehensive acoustic feature analysis^18^.

### Deep learning approaches and embeddings

Deep audio embeddings have been significantly improved in recent years, with i-vectors and x-vectors derived from deep neural networks showing superior performance in speaker identification and verification tasks^19^. X-vectors obtained from Time-delay Neural Networks (TDNN) capture speaker- discriminative characteristics in compact representations^9^. However, these methods operate as black-box feature representations that cannot be interpreted to understand vocal delivery characteristics. Sindhu et al. (2025) proposed Nested U-Net architectures with time-frequency attention for speech enhancement^20^. This represents an advancement in the field of signal processing; however, it does not explicitly support the purpose of speaker profiling. 3D dyadic motion data was generated from spoken text by Sun et al. (2025) using transformer-based models, capturing the dynamics of multimodal communication but without a taxonomic structure for vocal styles^21^. There is a gap in interpretability: the existing deep embeddings do not yield usable feedback relative to energy modulation, rhythmic patterns, or spectral clarity critical to coaches using this technology^8,9,11^.

### Research gaps and contributions

Table summarizes key limitations in prior work. The methods presently published are limited in their ability to produce interpretable embeddings for speaker identity discrimination whilst also providing little or no information regarding the delivery style’s contribution to emotion recognition (a) speaker identity discrimination with uninterpretable embeddings, (b) emotion recognition without delivery style granularity or (c) rhetorical analysis without acoustic grounding.Present studies address these gaps through the four contributions detailed in section 1(Novel Contributions).

## Proposed methodology

**Fig. 1:**
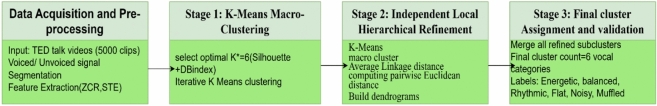
Overall workflow of the Proposed hybrid clustering framework.

The study introduces a novel hybrid clustering framework that combines K-Means macro clustering with Hierarchical Agglomerative refinement for scalable and interpretable speaker profilin. The three-stage method as explained in Fig. 1 consists of grouping speakers by applying K-Means initialization (k=6) methods first to group them into “Coarse” macro-clusters of speakers with similar vocal styles while reducing computation. The second step uses independent hierarchical clustering within each macro-cluster for locally classifying the speakers resulting in producing separate dendrograms with locally optimized cutting thresholds; The final step involves using a “Non-Uniform” Adaptive Subdivision technique using Silhouette score per macro-cluster. This technique is different from other clustering methods such as using a single global dendrogram or performing binary splits through uniform methods such as bisecting k means, as we successfully create six independent hierarchical trees (dendrograms) which maintain similar and therefore, more homogeneous sub-cluster divisions for each tree and, in addition, can further refine the heterogeneous nature of these categories of sub-cluster division. The computational efficiency (O(n²/k)) of this technique is greater than full hierarchical clustering techniques with respect to cluster cohesion and interpretation (O(n²)).

Stage 1: K-means macro-clustering partitions 500 speakers into 6 clusters. Stage 2: Independent hierarchical refinement builds separate dendrograms for each macro-cluster with adaptive cutting based on local silhouette optimization (shown as 6 separate trees). S3: Final cluster assignment combines refined subclusters into interpretable vocal categories.

## Experimental setup and reproducibility parameters

The experimental design of the TED speaker analysis framework is highly configured for full reproducibility, as it defines each of the hardware, software, data, pre-processing, feature extraction, and clustering parameters to be used in experiments. The experiments were conducted on a computer system equipped with an Intel i7 or AMD Ryzen 7 processor, 16 GB of RAM, and either Windows 11 or Ubuntu 22.04, and using MATLAB R2024a with Signal Processing and Machine Learning toolbox or optional Python libraries such as NumPy, SciPy, Librosa, and Scikit-learn. A comprehensive data set of 5,000 TED audio clips was selected from the official repository, with each audio clip converted from MP3 format to WAV format (16-bit resolution with a sample rate of 44.1 kHz) before proceeding with normalization after conversion to mono. Pre-processing involved down sampling to 44.1 kHz and pre-emphasis of each mono audio clip, which was subsequently framed, such that for every 25-millisecond window there was overlap with a new segment shifted by 10 milliseconds that would also replace the current segment^22,23^. A Hamming window was also applied to each audio clip, and the audio clips were peak-normalized. Voiced, unvoiced division was based on Zero Crossing Rate (threshold=0.12) and Short, Term Energy (threshold=0.025). The acoustic features extracted were ZCR, STE, Power Spectral Density via Welch’s method (512, sample window, 256, sample overlap, 1024, point FFT), Mean Power, FFT Magnitude, and Standard Deviation, all of which were normalized using Z, score scaling. Speaker clustering was a hybrid framework: firstly, K-means (K=6 k, means++ initialization, 300 iterations, 20 replicates) was used, and then Agglomerative Hierarchical clustering (average linkage, Euclidean distance, cophenetic threshold=0.40) was applied. The hybrid model had a silhouette score of 0.90 and thus was able to perform better than K, Means (0.52), Agglomerative (0.58), DBSCAN (0.55), and Spectral Clustering (0.60). Comparative performance across clustering methods was further validated using paired t-tests on bootstrap samples to ensure statistically rigorous comparisons. The optimal value of K=6 was determined using three different methods; the elbow method, silhouette analysis and the minimization of the DB index as described in Supplementary Figures S1 and S2, as well as Table S1. The ZCR/STE threshold values were determined by using the ROC curve optimization method on the pilot data (Supplementary Figure S3, Table S2).

### Hyperparameter optimization and threshold selection

By performing elbow analyses, maximizing silhouette scores and minimizing Davies-Bouldin indices, we determined the best K value (optimal K is best represented by an elbow within K = 2 - 10), as stated within Supplementary Figures S1-S2 and Supplementary Table S1. Using 500 pilot clips to optimize both the ZCR and STE thresholds (ZCR = 0.12 and STE = 0.025), we achieved 94.2% accuracy on voiced/unvoiced classifications when matched against expert annotations $(\kappa = 0.91)$. The results of the ROC analysis are also included in the Supplementary Figures S3 and Supplementary Table S2. Complete information regarding hyperparameter selection is contained in Supplementary Materials Section 1.

### Data description

The dataset utilized in this research entails 5,000 audio clips sourced from public TED Talks featured on the official TED website (https://www.ted.com/). The clips were chosen to ensure a representative sample, using three criteria: (i) topic diversity across technology, education, psychology, science, and communication; (ii) speaker diversity in gender, accent, and speaking style; and (iii) audio quality, assuring minimal artifacts and clips were 44.1 kHz. The dataset ultimately combines 500 unique speakers, encompassing 250 male presenters and 250 female presenters, and covering a wide range of English accents (American, British, Indian, Australian, and European). A detailed list of all TED speakers and talk titles included in the study is provided in the Appendix (Table: Sl. No, talk title, speaker name, TED talk URL)

Table 1 reports the summary of the dataset description. This dataset consists of 5,000 audio clips from 500 unique speakers, balanced equally by gender and representing diverse global English accent. Each recording spans 10–15 minutes, with high-quality audio selected across talks delivered. The TED Talk clips were sampled semi-randomly from TED Talks published between the years of 2010 - 2025, after filtering for clarity, topic diversity, and richness of content. All of the MP3 streams were converted to WAV given that it preserves the full-frequency information necessary to accurately calculate acoustic features such as the Zero Crossing Rate, Short-Term Energy, Power Spectral Density, and Magnitude.

**Table 1:** Summary of dataset description used in the study.

Attribute	Description
Total audio clips	5,000
Unique speakers	500
Gender distribution	250 male, 250 female
Duration range	10–15 minutes
Median clip duration	9 minutes
Accent diversity	American, British, Indian, Australian, European English
Date range of talks	2010–2025
Clips per speaker	10
Selection criteria	Audio quality, topic diversity, clarity, moderate background noise

### Signal processing foundations

To evaluate the speech characteristics, specific audio features are retrieved from the segmented WAV audio files from TED Talks. A signal processing system uses a fundamental property called ZCR (Zero Crossing Rate)^24,25^ to differentiate between the voiced-unvoiced portions of a speech signal. ZCR quantifies the rate at which audio signal changes sign, serving as a frequency-domain proxy for voiced/unvoiced discrimination^24,25^. The expression for this is shown in Eq. (1)

1 $$\begin{aligned} \textrm{ZCR}(f) = \frac{1}{L-1} \sum _{n=1}^{L-1} \textbf{1}\left[ s(n)\, s(n-1) < 0 \right] \end{aligned}$$

where L is the total number of trials in the frame, s(n) is the amplitude of the signal at time t, and 1 is an indicator function that is 1 if the condition is true and 0 otherwise.

**Fig. 2:**
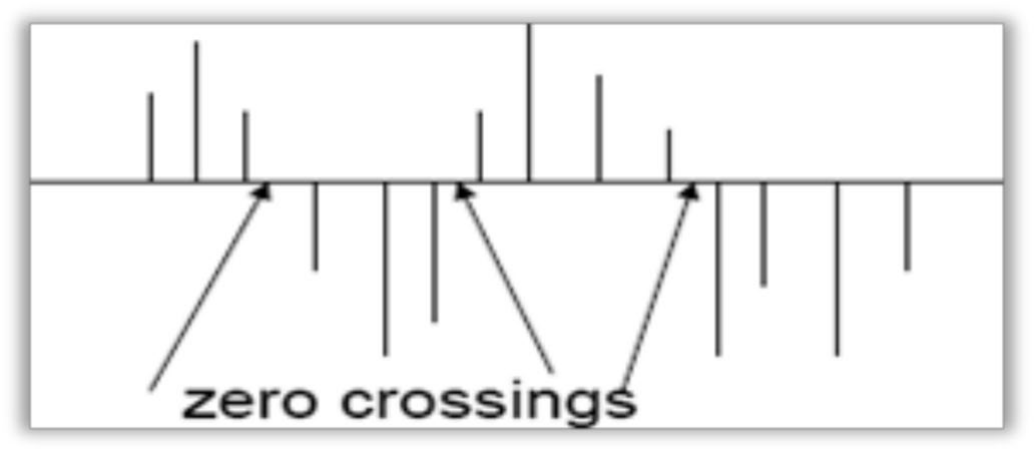
Illustration of zero crossings of a signal.

Zero crossing rate (ZCR): is mainly used in speech signal analysis to separate voiced-unvoiced signals. Voiced speech is periodic as vocal cords vibrate periodically when vowels are pronounced (a, e, i, o, u). In contrast, unvoiced speech is aperiodic, which includes noise utterances (hmmm, uhhhh) and pauses, resulting in higher ZCR. Zero crossings in audio signals are depicted as shown in Fig. 2.

Signal Discretization and Normalization: The continuous speech signal s(t) is sampled at frequency fs to obtain discrete samples [Eq. 2].

2 $$\begin{aligned} S[n] = s(t)\big |_{t = n T_s} \end{aligned}$$

where n is the sample index (0,1,2….),

$T_s$ is the sampling period $(Ts=1/ fs),$

fs is the sampling frequency.

Once the signal is sampled, it is divided into overlapping frames of length N. Each frame is normalized as shown in Eq. (3).

3 $$\begin{aligned} S_{\text {norm}}[n] = \frac{s[n]}{\max \left| s[n] \right| } \end{aligned}$$

s[n] is a speech sample in a frame, $S_{norm\left[ n\right] }$is the normalized amplitude, max(s[n]) is the maximum absolute value in the frame, which ensures all the frames are scaled to a uniform amplitude range typically 1 and −1, improving the feature extraction reliability. Computing the sign functions: Eq. (4) determines zero crossings where the sign changes from positive to negative and vice versa between consecutive samples as shown in Eq. (5)

4 $$\begin{aligned} \operatorname {sgn}(x[n]) = {\left\{ \begin{array}{ll} +1, & x[n]> 0, \\ 0, & x[n] = 0, \\ -1, & x[n] < 0. \end{array}\right. } \end{aligned}$$

x[n] is a single sample from a discrete-time audio signal, sgn(x[n]) sign of the sample tells us whether the sample is positive, negative, or zero as shown in Eq. (4).

5 $$\begin{aligned} \textbf{1}\!\left[ \operatorname {sgn}(x[n]) \ne \operatorname {sgn}(x[n-1])\right] \end{aligned}$$

**Fig. 3:**
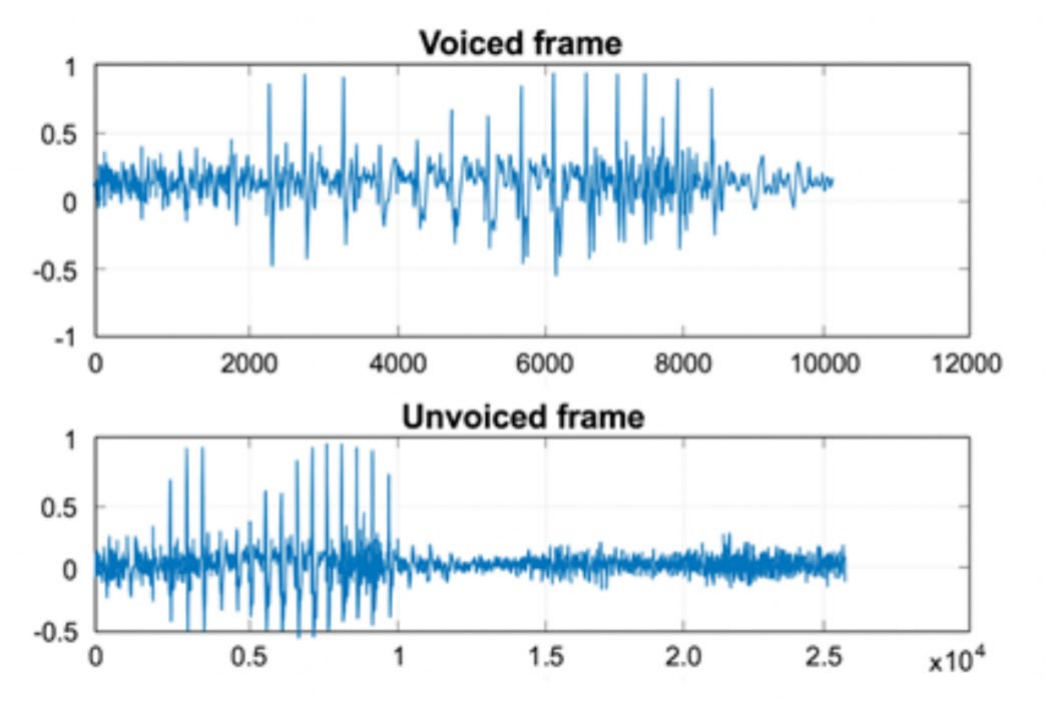
Illustration of zero crossings of a signal.

As shown in Fig. 3, voiced frames show periodic oscillations with fewer zero crossings, unvoiced frames show irregular amplitude changes with higher ZCR.

Short-Term Energy (STE) measures signal intensity within overlapping frames^5,24,25^. After applying the window function: Multiply each audio signal frame by a windowing function w(n) (Hamming window) as shown in Eq. (6). Frame energy $E_k$ is computed as given in Eq. 7.

6 $$\begin{aligned} x(n) = s(n)\, w(n) \end{aligned}$$

where w(n) is the window function, n is a discrete time index. Compute the Short-Term Energy: For each frame x(n), short-term energy is computed as

7 $$\begin{aligned} E_k = \sum _{n=0}^{N-1} x^2(n) \end{aligned}$$

$E_k$ is the energy of the k-th frame, N is the frame length, and x(n) is the windowed signal within the frame. Frame are shifted by 10–20 ms for temporal continuity. Classification: Voiced Speech: Frames with relatively high energy levels correspond to voiced speech segments. Unvoiced Speech: Frames with lower energy levels, often characterized by noise-like signals, may indicate unvoiced speech segments. Silence: Frames with minimal energy, typically representing silence or background noise.

**Fig. 4:**
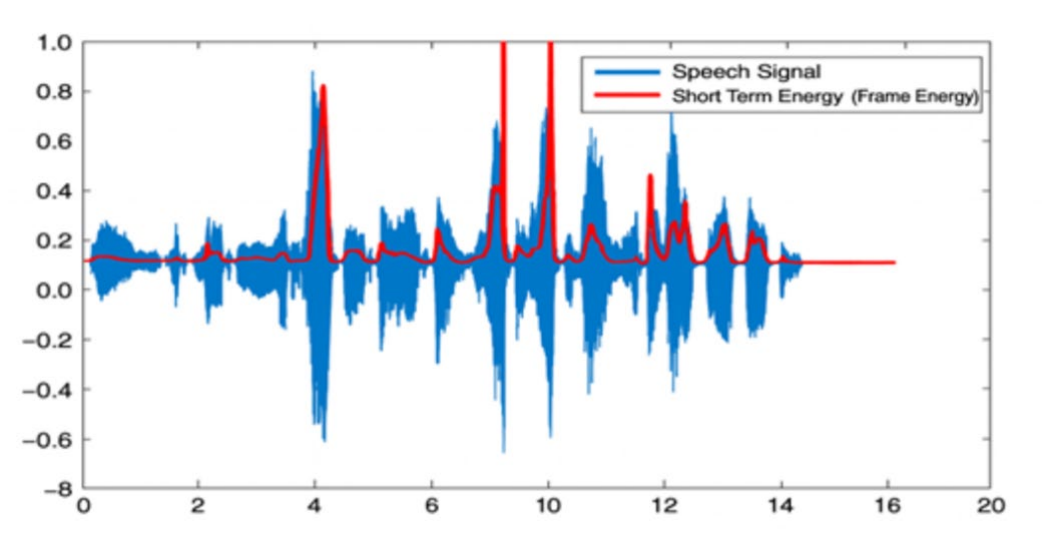
Short-Term Energy (STE) analysis of the speech Signal.

**Fig. 5:**
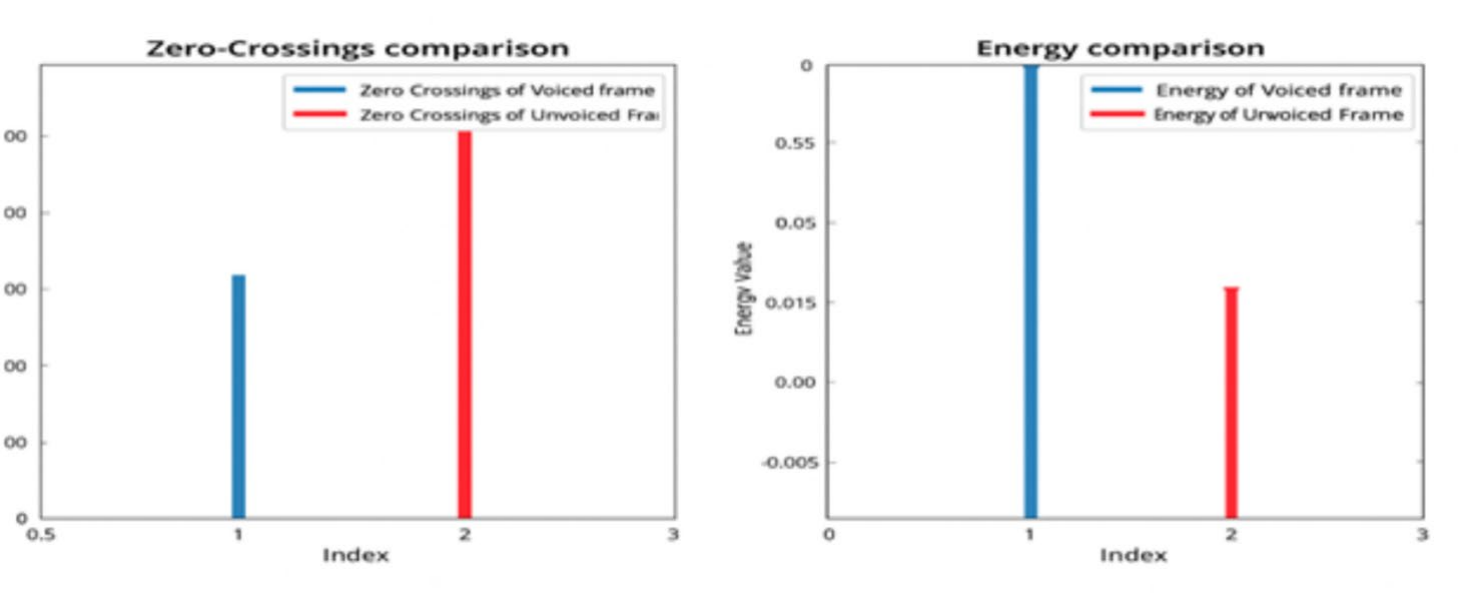
Comparison of ZCR and STE for voiced-unvoiced speech frames.

In this study, STE is used to analyze the energy level in the audio signal and to separate voiced-unvoiced signals from WAV audio files as shown in Fig. 4. The blue waveform represents the speech signal in the time domain, where change in amplitude values represents speech intensity over time. The red curve depicts Short-Term Energy (STE) calculated over audio frames, providing a refined representation of the speech signal energy over time. Peaks in the red curve indicate the high-energy regions of a speaker, typically associated with the voiced region of the speech. The low-energy regions represent unvoiced speech, where amplitude variations are small. Figure 5 highlights the complementary nature of ZCR and STE. ZCR discriminates based on spectral content (frequency).STE discriminates based on intensities (energy). voiced frames have fewer zero crossings, because they are periodic with lower frequency component. Unvoiced frames have higher zero crossings with frequent sign changes from positive to negative axis (vice versa), indicate high frequency components. Thus, ZCR clearly distinguish between voiced-unvoiced speech using ZCR.

### Audio feature extraction

As shown in Fig. 3, voiced frames show periodic oscillations with fewer zero crossings, unvoiced frames show irregular amplitude changes with higher ZCR. Specific audio features such as Power Spectral Density (PSD), Mean Power, Magnitude, and Standard Deviation are computed. These features represent the audio signal characteristics in compact form. Finally, the Hybrid clustering technique is applied to group similar audio segments for further analysis.

### Power spectral density

The Power Spectral Density (PSD) describes how the power signal is distributed across varying frequency components. PSD makes it possible to examine the frequency content of audio signals, providing crucial details regarding the energy distribution across various frequency bands^26^. PSD analysis helps novice TED speakers understand their vocal delivery and how energy is distributed across different energy bands during their speech. In TED Talks, effective speakers show balanced energy distribution in their speech with clear voice articulation in both low and high frequencies, reflecting their vocal clarity. By comparing PSD patterns, novice speakers can vary their articulation, pitch variation for acoustically well-modulated speech. Power spectral density (PSD) s(f) of a signal x(t) is given by Eq. (8), which indicates how the power of a signal is distributed across various frequencies

8 $$\begin{aligned} S(f) = \lim _{T \rightarrow \infty } \frac{1}{T} \left| X_T(f) \right| ^2 \end{aligned}$$

$\left( X_T(f)\right)$ is the Fourier transform of the finite-time signal. T is the Observation window length, ${\ |\left( X_T(f)\right) \ |}^2$ is the squared magnitude of the frequency domain. The Fourier transform of the time-domain signal x(t) is defined in Eq. (9).

9 $$\begin{aligned} X(f) = \int _{-\infty }^{\infty } x(t)\, e^{-j 2\pi f t}\, dt \end{aligned}$$

Where $X\left( f\right)$ is the frequency domain represention of the signal $x\left( t\right) . x\left( t\right)$ is the time domain signal. $e^{-j2\pi ft}dt$ is the complex sinusoid is used to decompose the signal.

Unvoiced speech can be mathematically modelled as a wide-sense stationary random process (WSS). Autocorrelation depicts a measure of how much the signal repeats or resembles itself over time. Defining the autocorrelation function $R_x\left( \tau \right)$ for the unvoiced speech signal as indicated in Eq. (10) $E[x\left( t\right) ]$ is the expectation. $\tau$ is lag or delay $x\left( t\right) .x\left( t+\tau \right)$ are the signal values at time t and a later time $t+\tau .$

10 $$\begin{aligned} R_x(\tau ) = E\!\left[ x(t) *x(t+\tau ) \right] \end{aligned}$$

Autocorrelation function for unvoiced as given in Eq. (11). $\sigma ^2$ is variance of the white noise signal. $delta(\tau )$ the Dirac delta function has a value at $\tau =0$. The autocorrelation of unvoiced speech is 0 at all non-zero delays.

11 $$\begin{aligned} R_x(k) = {\left\{ \begin{array}{ll} \sigma ^2, & k = 0, \\ 0, & k \ne 0 \end{array}\right. } \qquad R_x(\tau ) = \sigma ^2 \delta (\tau ) \end{aligned}$$

It matches the non-periodic random nature of unvoiced speech. Link to PSD using Wiener-Khinchin theorem. Applying the Wiener-Khinchin theorem, as given in Eq. (12), relates the PSD of a signal to the Fourier transform of its correlation.

12 $$\begin{aligned} S(f) = \int _{-\infty }^{\infty } R_x(\tau )\, e^{-j 2\pi f \tau }\, d\tau \end{aligned}$$

Function for the eradication of white noise with autocorrelation is given in Eq. (13)

13 $$\begin{aligned} R_x(\tau ) = \sigma ^2 \delta (\tau ),\qquad S(f) = \int _{-\infty }^{\infty } \sigma ^2 \delta (\tau )\, e^{-j 2\pi f \tau }\, d\tau \end{aligned}$$

**Fig. 6:**
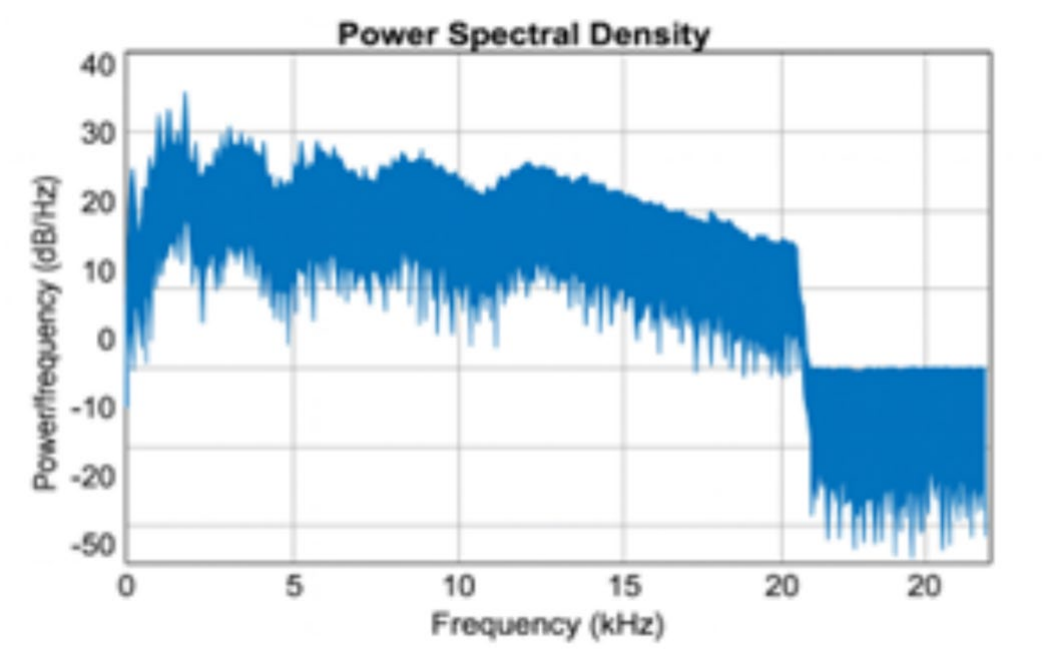
Power spectral density plot for a given speech signal.

**Fig. 7:**
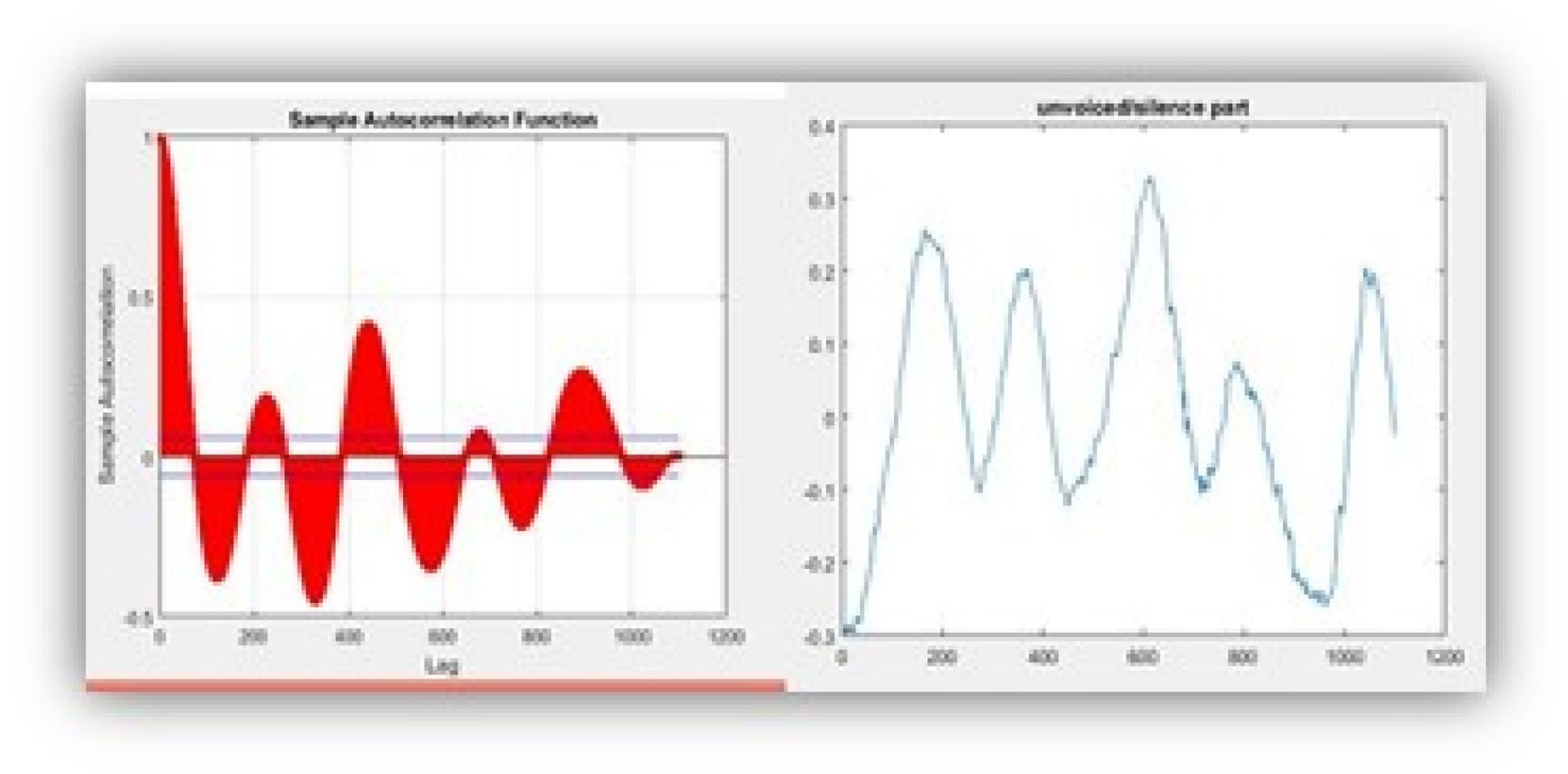
Autocorrelation segment in speech.

The PSD plot in Fig. 6 shows the spectral energy distribution across frequencies, showing higher power at lower frequencies with gradual decline at increasing frequency. The sharp decrease near 20 kHz low-pass filter effect indicates a filtering effect with small fluctuations, suggesting the presence of random noise in the signal.

Figure 7 is a sample Autocorrelation function (ACF) to identify the periodicity and pitch. It also shows the waveform of the unvoiced part of the speech signal, typically marked by low amplitude. Representative features are calculated from the TED speakers’ speech for both voiced-unvoiced signals. Autocorrelation and signal plots of unvoiced segments help us evaluate TED speakers’ voice modulation, pause quality, rhythm -all of which are strong indicators of effective communication. Good speakers exhibit structured unvoiced patterns, as unvoiced speech carries essential acoustic information, purposeful pauses indicating confidence and clarity in the speech. These features are crucial for distinguishing effective speakers from novice speakers.

### Mean power (intensity) in air

Mean power is an acoustic feature reflecting speaking style, clarity, and individual traits. Mean Power is essential for TED speaker classification because it enables recognition of speech dynamics such as, high-energy, calm, and soft-spoken speakers. High-energy speakers usually show higher mean power and a larger variation. Calm speakers show lower mean power. The mean power of a speech signal measures the average energy of the waveform over a time window as shown in Eq. (16). For speech signal x[n]:

14 $$\begin{aligned} P_{\text {mean}} = \frac{1}{N} \sum _{n=0}^{N-1} x^2[n] \end{aligned}$$

where Higher values indicate stronger and louder speech, and Lower values indicate softer and quieter speech.

### Magnitude in audio signal

Magnitude refers to the strength or loudness of a given signal. In digital signal processing, magnitude refers to the amplitude of a signal, and it is used to measure how strong the signal is at a given sample. If x[n] is the discrete audio signal, then the mean magnitude can be defined over N samples as

15 $$\begin{aligned} \text {Mean Magnitude} = \frac{1}{N} \sum _{n=0}^{N-1} \left| x[n] \right| \end{aligned}$$

Equation (17) is used to estimate average signal strength. While both magnitude and mean power reflect speech strength, mean power is more sensitive to emotional intensity and expressive speaking style, making it a more robust for classifying TED speakers based on delivery energy and emphasis. Magnitude, being a more conservative average of signal amplitude, is better suited for identifying silence vs. speech regions in the audio signal^7,27^.

Mathematical steps for extracting the magnitude spectrum from an audio signal in a concise format are as follows: In the first step, framing of the signal is done, where the audio signal is split into overlapping frames of length N as represented in Eq. (18).

16 $$\begin{aligned} x_k[n], \quad n = 0,1,2,\ldots , N-1 \end{aligned}$$

In the next step windowing function is applied to every frame by multiplying each frame $x_k [n]$. By a window function w[n] to get $x_w [n]$ as given in Eq. (19).

17 $$\begin{aligned} x_w[n] = x_k[n] \, w[n] \end{aligned}$$

Computing the relevant time-domain features from the windowed signal as shown in Eq. (20).

18 $$\begin{aligned} x_w[n] \end{aligned}$$

Computing the magnitude spectrum by taking the absolute value of the complex FFT output to obtain the magnitude spectrum as given in Eq. (21).

19 $$\begin{aligned} \left| x_w[k] \right| = \sqrt{ \bigl (\operatorname {Re}\{x_w[k]\}\bigr )^2 + \bigl (\operatorname {Im}\{x_w[k]\}\bigr )^2 } \end{aligned}$$

Magnitude spectrum:$M_k\left[ m\right] =|x_w\ [m]|$, where $M_k\left[ m\right]$ represents the frequency content of the signal for frame k, suitable for feature extraction in the audio signal

### Standard deviation (SD)

Standard deviation is a statistical measure that quantifies amplitude variation values into an audio signal over time. For a discrete audio signal, x[n], with N samples, we first compute the mean$\mu$,

20 $$\begin{aligned} \mu = \frac{1}{N} \sum _{n=0}^{N-1} x[n] \end{aligned}$$

the standard deviation is then calculated as shown in Eq. (23).

21 $$\begin{aligned} \text {Standard Deviation (SD)} = \sqrt{\frac{1}{N} \sum _{n=0}^{N-1} \left( x[n] - \mu \right) ^2} \end{aligned}$$

**Table 2:** Statistical summary of extracted time-domain features.

Feature	Mean	SD	Min	Max	Time Complexity
Short-Time Energy (STE, normalized)	0.0318	0.0051	0.02	0.05	*O*(*N*)
Zero Crossing Rate (ZCR, crossings/frame)	132.4	12.8	108	159	*O*(*N*)
Mean Power (dB)	0.587	0.041	0.48	0.68	*O*(*N*)
Mean Magnitude	0.891	0.018	0.85	0.93	*O*(*N*)
Standard Deviation (SD)	0.124	0.012	0.10	0.15	*O*(*N*)

Table 2 presents acoustic feature metrics for TED Speaker Delivery analysis. The 5,000-clip dataset displays stable acoustic trends across speaker. STE values indicate high consistent energy distribution, while mean power and magnitude differentiate high-energy from calm speakers. The standard deviation remains narrow (0.124 ± 0.012), representing controlled within- speaker variability.

### Classification

In the classification stage, each TED speakers’ voice is converted into a five-dimensional feature vector based on features such as ZCR, STE, Mean Power, Magnitude, and Standard Deviation. The proposed Hybrid Clustering applies K-means first to group thousands of audio data clips to group similar speech styles into compact clusters. we then apply Hierarchical clustering within these clusters for fine-grained classification. This Hybrid approach balances computational efficiency with analytical depth, making it both practical and effective for analyzing thousands of TED speaker profiles.

We initially applied Agglomerative hierarchical clustering to group TED speakers by vocal traits. However, we found that although it uncovers detailed patterns, it performs poorly on large datasets due to its computationally expense:$O (n^3)$ time complexity and $O (n^2)$ space complexity. Moreover, once clusters are merged in early stages, they cannot be separated, which often leads to suboptimal results, especially with noisy data. To address these challenges, we adopted a hybrid method: first using K-means to create broad clusters, followed by Hierarchical clustering to capture finer vocal nuances more effectively.

### Agglomerative hierarchical clustering model for TED speaker classification

Agglomerative hierarchical clustering^7,9^, is a unsupervised learning technique well-suited for analyzing structured patterns in audio features, particularly when categories are not predefined-a common scenario with real-world speech data such as TED Talks. In this study, we adopt an Agglomerative hierarchical clustering to classify TED speakers based on their acoustic characteristics derived from features such as Zero Crossing rate (ZCR), short-term energy (STE), Power spectral density, mean Power, standard deviation, and magnitude. Hierarchical clustering^8,11^, is an unsupervised learning method that constructs a hierarchical tree(dendrogram) by successive merging individual data points into larger clusters based on their similarity. TED Talks features diverse speakers, with varying speaking styles, emotions, pitch, accent, and pauses. We often lack labelled data for these features in real time, making supervised methods impractical. Hence, unsupervised learning is necessary to discover natural grouping (energetic Vs calm speakers, high-pitched Vs low-pitched speakers). We propose a clustering method that naturally accommodates temporal audio data, allows progressive merging of similar fragments and does not require predefining the number of speech patterns. The output is a dendrogram, that visualizes cluster relationship.

The general Agglomerative hierarchical clustering^28^ is as defined below. Let $X_i\in R^d$ represents the feature vector of the *ith* speaker, where d is the number of features. The Euclidean distance between two feature vectors $X_i \; \text {and} \; X_j$ is given as in Eq. (24).

22 $$\begin{aligned} D(X_i, X_j) = \sqrt{\sum _{k=1}^{d} \left( x_{i,k} - x_{j,k} \right) ^2} \end{aligned}$$

To construct the cluster hierarchy, we use average linkage criteria, which define the distance between two clusters $C_p \; \text {and} \; C_q$ as the average pairwise distance between all elements of the clusters, as given in Eq.(25).

23 $$\begin{aligned} D(C_p, C_q) = \frac{1}{|C_p |\, |C_q |} \sum _{X_i \in C_p} \sum _{X_j \in C_q} D(X_i, X_j) \end{aligned}$$

The clustering process begins with each speaker as a singleton cluster. At each iteration, 2 clusters with the smallest average linkage distance are merged. This process continues until all data points are grouped into a single cluster, forming a dendrogram that represents the nested groupings of speakers and their corresponding similarity levels. To determine the optimal number of speaker clusters, we analyze the dendrogram structure using quantitative validation methods such as the silhouette score as given in Eq. (26).

24 $$\begin{aligned} \text {Silhouette Score} = \frac{b(i) - a(i)}{\max \{a(i),\, b(i)\}} \end{aligned}$$

a(i) average distance between point i and all other points in the same cluster. b(i) is the lowest average distance between point i and all points in the next nearest cluster. This method enables the effective categorization of TED speakers based on vocal traits, offering insights into the speech delivery patterns and energy levels of speakers. Distance metric calculation using Manhattan distance as represented in Eq. (27).

25 $$\begin{aligned} d(C_i, C_j) = 1 - \sum _{k=1}^{p} |x_{ik} - x_{jk} |\end{aligned}$$

The covariance $cov (C_iC_j)$ is the covariance between the clusters $C_iC_j$ and their variances are $var(C_i )$ $var(C_j)$ and their variances. After merging clusters $C_iC_j$ in to $C_k$ update the dissimilarity matrix D to reflect the new distances.

26 $$\begin{aligned} d(C_k, C_m) = \text {linkage}(C_k, C_m) \end{aligned}$$

Where the linkage function determines how the distance between clusters $C_kC_m$ is calculated after merging as given in Eq. (28).

**Fig. 8:**
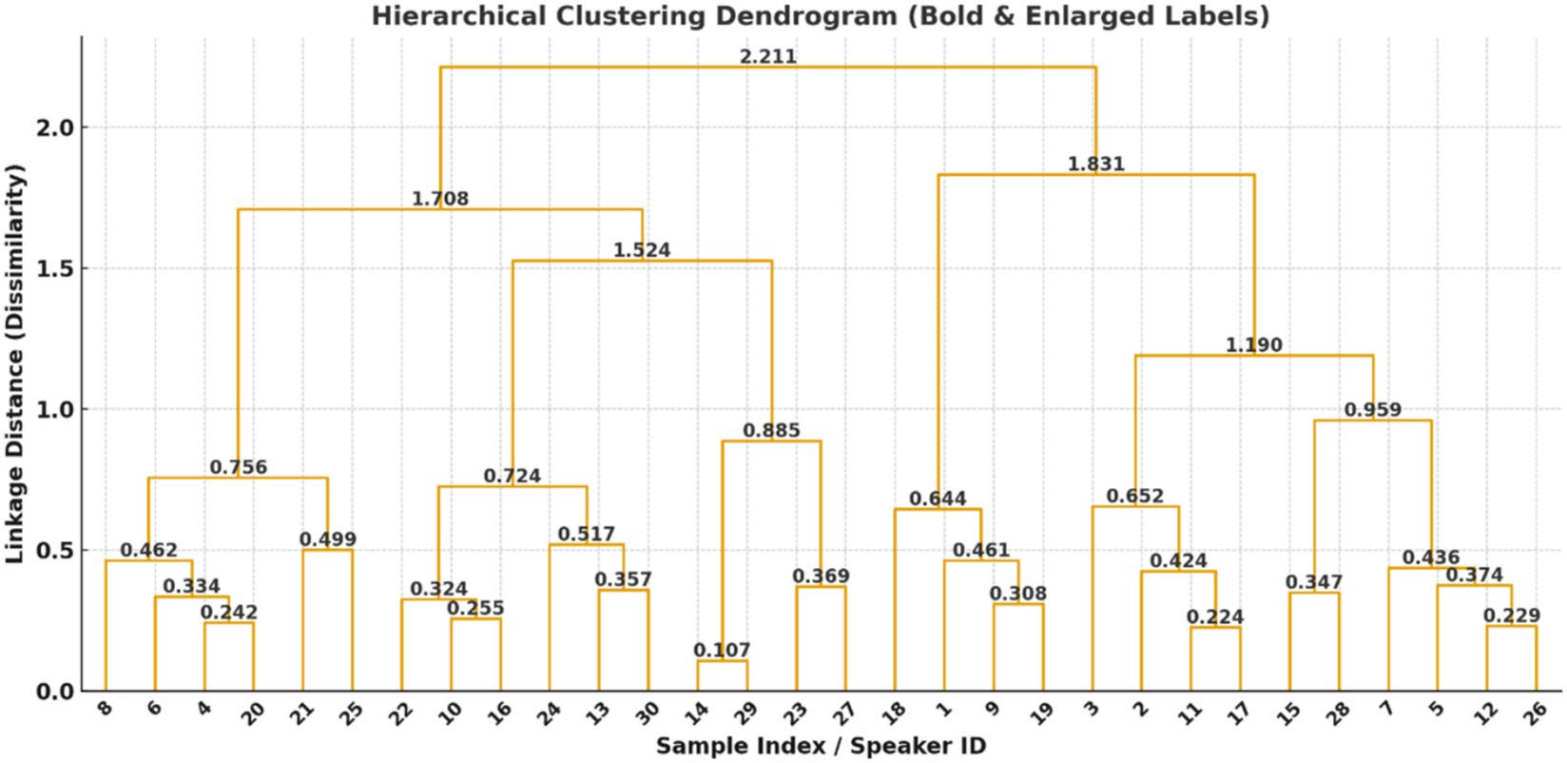
Dendrogram binary cluster tree.

Figure 8 shows the resulting Dendrogram binary tree clusters for selected speakers. The dendrogram visualizes how speakers are merged step-by-step based on similarity, with lower linkage distances indicating more similar speech traits^2,29^. Higher branches show less similar speakers, and the displayed merge distances help interpret cluster strength and separation. But Agglomerative hierarchical clustering is slow for large data, $O (n^3)$ time and, $O (n^2)$ space, and it is irreversible as it merges data in the early stages. This method is not robust to noisy features. A dendrogram gets messy with many data points. Hence, we have designed a novel hybrid method which combines k-means and Hierarchical method, where k-means pre-groups similar data, reducing early poor merges and filtering noise in pre-grouping. The dendrogram is retained and less cluttered. TED speakers vary across several vocal dimensions like energy, pitch, tone, and pauses. These form thousands of feature vectors. The hybrid method segments a large group first using k-means-like categorizing broad range of vocal styles. (calm and energetic speakers). Then we apply fine-grained hierarchy to capture nuances (enthusiastic Vs persuasive tone). This two-level granularity (broad and specific) makes speaker style discovery natural and interpretable compared to already existing methods like a monolithic hierarchical tree. We prefer the hybrid method as we are working on thousands of audio samples of TED speakers and dealing with complex vocal features. We are interested in both coarse and fine categorization of speakers. The hybrid clustering method will establish each TED speaker as a five-dimensional feature vector of Zero Crossing Rate (ZCR), Short-Term Energy (STE), Mean Power, Magnitude, and Standard Deviation, which will be normalized using Z-score normalization to establish the feature space. K-means clustering^30^, will be utilized to cluster together similar speech profiles as compact clusters, whereby data points are repeatedly assigned to the closest centroids, and these centroids are then updated for convergence. Agglomerative hierarchical clustering will be used in or between the K-means clusters to refine clusters further, identifying more details or closeness within clusters using a distance-based merge strategy^31,32^. The created clusters will be evaluated for quality and coherence using the Silhouette Score algorithm to assess separation for clusters and establish the ideal cluster structure. The hybrid clustering method provides an accurate, interpretable, and robust solution to speaker profiling. The hybrid method first identifies compact clusters with K-means, and then uses hierarchical clustering to refine the compact clusters, providing both scalability and detailed separation^29^.. Our goal is to cluster TED speakers based on voice delivery traits using ZCR (Zero Crossing Rate)-speech sharpness, short-term energy (STE)-speech energy over time, mean Power-overall intensity, standard deviation-variability in speech signal, and magnitude-amplitude^33–35^,

## Proposed hybrid hierarchical clustering based-K-means model (HC-KM)

This research presents a Hybrid Hierarchical-K-means framework that combines k-means efficiency with hierarchical sensitivity. Unlike bisecting K-Means (uniform binary splits) or global hierarchical methods (single dendrogram), our approach builds six independent dendrograms-one per K-means macro clusters -with locally optimized cutting thresholds enabling adaptive subdivisions (m * 1–6 subclusters per macro -cluster). For each macro-cluster Mj, the number of subclusters m* is selected from the candidate range 1, 2, 3, 4, 5, 6 by maximizing the local silhouette score computed exclusively within Mj. The upper bound of 6 is a per-cluster maximum (not a global constraint across all macro-clusters), empirically justified by silhouette score saturation observed beyond m = 6 in preliminary experiments and by the interpretability limit beyond which subcluster categories became acoustically indistinct. Subdivision halts early if the silhouette gain between successive cuts is below a threshold of 0.01, allowing homogeneous macro-clusters to remain as a single cluster (m* = 1) without forced splitting. All extracted features (ZCR, STE, PSD, Mean Power, Magnitude, Standard Deviation) were standardized using Z-score normalization before 3-stage process: K-means macro-clustering (k=6), independent hierarchical refinement, validated cluster assignment

### Algorithm 1: Hybrid Hierarchical-K-means for TED Speaker Profiling

**Input:** Feature matrix *X*, where each TED speaker is represented as a vector (Eq. 27):

27 $$\begin{aligned} X_i = [\textrm{ZCR}_i, \textrm{STE}_i, \textrm{MeanPower}_i, \textrm{Magnitude}_i, \textrm{StdDev}_i] \in \mathbb {R}^5 \end{aligned}$$

**Initialization:** Normalizing each feature dimension using Z-score (Eq. 28):

28 $$\begin{aligned} X_{i,k}^\mathrm{(norm)} = \frac{X_{i,k} - \mu _k}{\sigma _k} \end{aligned}$$

**Step 1:** Initialize *K* centroids randomly:

$$\mu _1, \mu _2, \dots , \mu _K, \quad C_j \text { is the cluster list.}$$

**Step 2:** Repeat until convergence:

1. **Assign each point** $X_i$ **to the nearest centroid:**
  29 $$\begin{aligned} C_j = \left\{ X_i \,\Big |\, j = \arg \min _{j} \Vert X_i - \mu _j \Vert ^2 \right\} \end{aligned}$$
2. **Update centroids:**
  30 $$\begin{aligned} \mu _j = \frac{1}{|C_j|} \sum _{X_i \in C_j} X_i \end{aligned}$$

**Step 3:** Repeat Eqs. (29) and (30) until cluster centroids converge.

**Output:** *K* compact clusters $C_1, C_2, \dots , C_K$.

### Stage 1: K-means macro-clustering

**Step 1.1: Determine the optimal number of clusters**

Test $k = 2$ to 10 using:

- Elbow curve (sum of squared errors)
- Mean Silhouette score
- Davies-Bouldin index

Optimal number of clusters: $k^* = 6$.

**Step 1.2: Initialize** *k* **centroids**

Randomly initialize $k=6$ centroids $\mu _1, \dots , \mu _6$ using K-means++ initialization. Set $C_j$ as cluster list for $j=1$ to *k*.

**Step 1.3: Iterative K-means clustering**

While not converged (centroid shift $< \epsilon < 10^{-4}$) and iterations $< 300$:

1. **Assign each speaker** $X_i$ **to the nearest centroid**:

31 $$\begin{aligned} C_j = \left\{ X_i \,\Big |\, j = \arg \min _j \Vert X_i - \mu _j \Vert ^2 \right\} \end{aligned}$$

2. **Update centroids:**

32 $$\begin{aligned} \mu _j = \frac{1}{|C_j|} \sum _{X_i \in C_j} X_i \end{aligned}$$

**Output of Stage 1:** Six macro-clusters $M_1, M_2, M_3, M_4, M_5, M_6$.

### Stage 2: Independent local hierarchical refinement

For each K-means macro-cluster $M_j$, $j = 1, \dots , 6$, perform independent hierarchical clustering:

**Step 2.1: Extract speakers in current macro-cluster**

33 $$\begin{aligned} X_j = \{ X_i \in X \,|\, X_i \in M_j \} \end{aligned}$$

**Step 2.2: Compute pairwise distances using average linkage**

34 $$\begin{aligned} D(C_p, C_q) = \frac{1}{|C_p| \, |C_q|} \sum _{X_i \in C_p} \sum _{X_j \in C_q} \Vert X_i - X_j \Vert ^2 \end{aligned}$$

Build dendrograms by merging clusters ($C_p, C_q$)

Step 2.3: Evaluate the cuts for $m \in \{1,2,3,4,5,6\}$ subclusters within macro-cluster $M_j$. Note: 6 is a per-cluster upper bound (not a global constraint), justified empirically by silhouette score saturation beyond $m=6$ in preliminary experiments and by the interpretability limit of acoustically meaningful categories.
Step 2.4: Compute local silhouette score *S*(*m*) for each candidate *m*. Apply early stopping: if $S(m) - S(m-1) < 0.01$, do not increment *m* further.
Step 2.5: Select
  $$m^{*} = \arg \max \{S(m) : m \in \{1,\dots ,6\}\}$$
  subject to the early stopping condition above. If $M_j$ is homogeneous, this naturally yields $m^{*}=1$ (no sub-division). If $M_j$ is heterogeneous, $m^{*}$ reflects the level of internal structure. Update the cluster list until the desired number of final clusters is reached.

### Stage 3: final cluster assignment and validation

The combined subclusters provide the final cluster labels, forming six acoustic categories. Clustering quality is validated using:

- Overall Silhouette score
- Davies-Bouldin index

**Silhouette score for each TED speaker** $X_i$**:**

35 $$\begin{aligned} s(i) = \frac{b(i) - a(i)}{\max \{a(i), b(i)\}} \end{aligned}$$

where:

- *a*(*i*): average intra-cluster distance of $X_i$
- *b*(*i*): average nearest-cluster distance of $X_i$

**Output:**

- Final set of refined clusters $C_\textrm{final}$
- Silhouette score *s*(*i*) for each TED speaker instance $X_i$

**Cluster labels:** Energetic, Balanced, Rhythmic, Flat, Noisy, Muffled.

**Table 3:** Hyperparameter summary.

Parameter	Value
K-means initialization	K-means++
K-means replicates	20
Maximum iterations	300
Tested *K* range	2–10
Selected $K^*$	6
Distance metric	Euclidean
Hierarchical linkage	Average
Local subcluster range	1–6 per macro cluster (per-cluster max; early stop if gain < 0.01)
Final number of clusters	6

**Table 4:** Comparison of clustering subdivision strategies.

Method	Splitting Strategy	Final Count	Key Difference	Our Innovation
Bisecting K-means	Uniform binary	$2^{\text {depth}}$	Forces equal splits	We use 6 separate dendrograms with local optimization
Two-Level K-means	K-means at both stages	$K_1 \times K_2$ $K_1$: clusters in stage 1 $K_2$: clusters in stage 2	No hierarchy	We add hierarchical structure per cluster
Global Hierarchical	Single dendrogram	Cut at threshold	$O(N^2)$	We use $O(N^2 / K)$ per cluster with independent cutting
Proposed Method	Independent per cluster	Silhouette-optimized	Adaptive non-uniform subdivision	Builds 6 independent dendrograms; each cut selects m* $\in$ 1–6 subclusters per macro-cluster (per-cluster max; early stop if silhouette gain < 0.01).

Table 3 summarizes the essential hyperparameters incorporated into the proposed hybrid approach, including the initialization for K-means, the chosen $\kappa *$, the hierarchical linkage type, and the local refinement parameters. These parameters contribute toward formulation reproducibility and explanation of the clustering framework used in the current study. Table 4 compares four clustering subdivision strategies based on their splitting approach. While existing methods use uniform splits (bisecting- K-means), non-hierarchical structures (two-level K-means), or computationally expensive global dendrograms (hierarchical clustering), the proposed method performs independent, adaptive subdivision of each macro-cluster using silhouette-score optimization.

While hierarchical clustering is applied independently within each macro-cluster, not globally. Each macro-cluster receives its own dendrogram with its own optimal cutting threshold, allowing non uniform subdivision (e.g. $M_2$ remains intact while $M_1$ splits into 2). This differs fundamentally from bisecting K-means (forces binary splits everywhere) and global hierarchical methods (single dendrogram for all 500 speakers).

### Computational complexity

- **Stage 1: K-means Macro-Clustering**
  $$O(N \times K \times D \times I) \approx 500\,\text {k operations} \quad (N=500,\, K=6,\, D=5,\, I=50)$$
- **Stage 2: Local Hierarchical Refinement**
  $$O\Bigg (\sum _j N_j^2 \times D\Bigg ) \approx 312\,\text {k operations} \quad \text {(4 clusters} \times 125^2 \times 5\text {)}$$
- **Total:**
  $$O(812\,\text {k}) \quad \text {vs. } O(1.25\,\text {M}) \text { for full hierarchical clustering} \quad \Rightarrow 1.54\times \text {faster}$$

## Results and discussion

This section presents the outcomes of the proposed hybrid clustering framework, effectively categorizes TED speakers into meaningful vocal profiles by identifying distinct vocal delivery patterns. The dataset comprises of 5,000 TED Talk audio clips from 500 speakers, providing robust foundation for understanding how vocal delivery variables cluster naturally into distinct speaking styles. Using the proposed hybrid K-means and Hierarchical Clustering approach, we clustered data into six acoustically meaningful clusters: Balanced, Flat, Energetic, Rhythmic, Noisy, and Muffled. These clusters demonstrates consistency across the dataset, indicating that the model is stable in recognizing similar acoustic patterns across an expanded speaker corpus.

**Table 5:** Summary statistics of acoustic features and cluster assignments computed over the full dataset of 5,000 TED talk audio clips.

Cluster	n (clips)	ZCR (mean ± SD)	STE (mean ± SD)	Mean Power (mean ± SD)	Magnitude (mean ± SD)	Std. Dev. (mean ± SD)	Label
A	1200	0.142 ± 0.021	0.058 ± 0.009	0.61 ± 0.07	0.74 ± 0.06	0.14 ± 0.02	Balanced
B	900	0.089 ± 0.014	0.031 ± 0.006	0.34 ± 0.05	0.41 ± 0.05	0.07 ± 0.01	Flat
C	1000	0.185 ± 0.028	0.094 ± 0.011	0.83 ± 0.09	0.93 ± 0.07	0.18 ± 0.03	Energetic
D	800	0.158 ± 0.020	0.067 ± 0.008	0.55 ± 0.08	0.69 ± 0.09	0.21 ± 0.02	Rhythmic
E	600	0.231 ± 0.039	0.028 ± 0.007	0.29 ± 0.04	0.50 ± 0.06	0.25 ± 0.03	Noisy
F	500	0.071 ± 0.012	0.022 ± 0.005	0.25 ± 0.03	0.38 ± 0.04	0.06 ± 0.01	Muffled
Overall	5000	0.146 ± 0.041	0.050 ± 0.027	0.48 ± 0.23	0.61 ± 0.28	0.15 ± 0.08	–

Note: Std. Dev.= Standard deviation of the audio signal amplitude.

Table 5 shows the average values along with the standard deviations of acoustic features (ZCR, STE, Mean Power, Magnitude, Standard Deviation) for six clusters that account for the most considerable differences in energy, spectral content, and temporal variability. The Energetic cluster has indeed the highest STE and Mean Power, whereas the Muffled and Flat clusters indicate the lowest values, thus, the different vocal, style profiles have been confirmed. PCA demonstrated that the first two components accounted for more than 80% of the variance, thus, they could be considered as vocal energy and temporal, spectral modulation axes. The large standard deviations in the “Overall” row (for instance, Mean Power 0.48 ±0.23) indicate that there was a great deal of acoustic variation in the whole dataset, which included six different vocal styles from Muffled (0.25±0.03) to Energetic (0.83 ±0.09). Such a wide variance between the clusters is the reason why the clustering method was used, as each cluster has a much lower variability within the cluster, thus the vocal subgroups that are homogeneous have been found successfully.

**Fig. 9:**
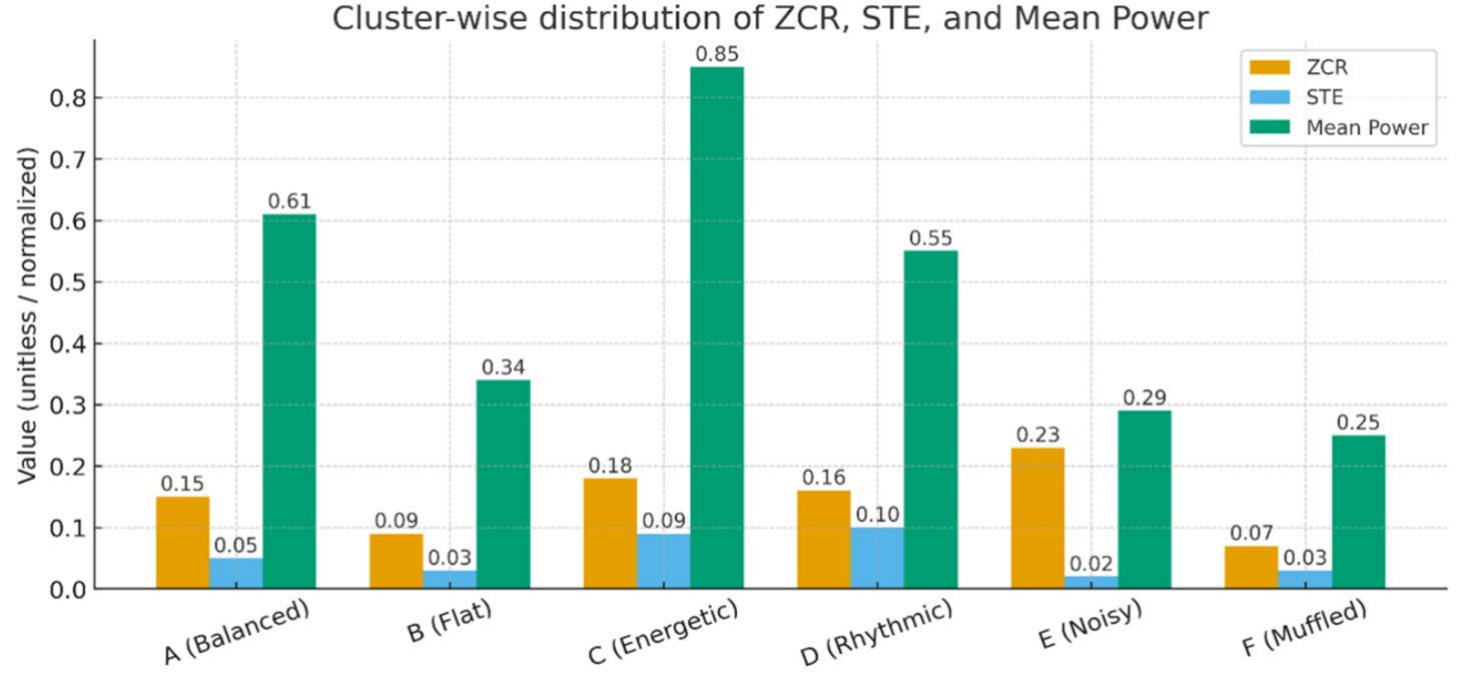
Plot of cluster-wise distribution of ZCR, STE, and Mean Power across 5,000 TED audio clips.

Figure 9 clearly shows ZCR, STE, and Mean power, vary across the six speaker style clusters, highlighting the strong acoustic separation. Energetic and rhythmic speakers show the highest energy levels, whereas flat and muffled categories exhibit the consistently lower spectral and temporal activity across all features.

### Internal validation

Principal component analysis is a dimensionality reduction technique that helps us simplify high-dimensional data by reducing the data to two or three main components (PCA1 and PCA2). PCA reduces multiple audio features (ZCT, STE, SD, PSD) into main key components for easier visualization. PCA1 captures the most important information, like direction and variance of audio features. PCA 2 captures the second most variance, orthogonal to PCA1. By plotting speakers in the PCA space, we can see which speakers have similar distinct audio characteristics. PCA scatter plot separates clusters A, B, C, and D, indicating distinct vocal feature groupings. Dendrograms represent the hierarchical merging of clusters using the hybrid approach. Detailed PCA loadings, hierarchical refinement parameters are explained in supplementary document. (Table S7, Table S11, Figure S12, Table S11, Table S12) Figure 12 depicts the average linkage distance within each coarse K-means cluster, showing the stable compact grouping prior to hierarchical refinement. Lower distances (0.136)-cluster 2 confirm that sub-clusters were formed from well-defined acoustic features and it is more compact and higher value (0.169)- cluster 3 indicates that greater internal variability and looser groupings.

**Table 6:** Silhouette score distribution.

Cluster	Category	Silhouette Score (Mean ± SD)	95% CI
A	Balanced	0.82 ± 0.04	[0.79–0.86]
B	Flat	0.88 ± 0.03	[0.84–0.91]
C	Energetic	0.91 ± 0.02	[0.88–0.93]
D	Rhythmic	0.87 ± 0.03	[0.83–0.90]
E	Noisy	0.69 ± 0.07	[0.61–0.76]
F	Muffled	0.72 ± 0.05	[0.66–0.78]
Overall	–	0.90 ± 0.02	[0.87–0.92]

**Figure 10:**
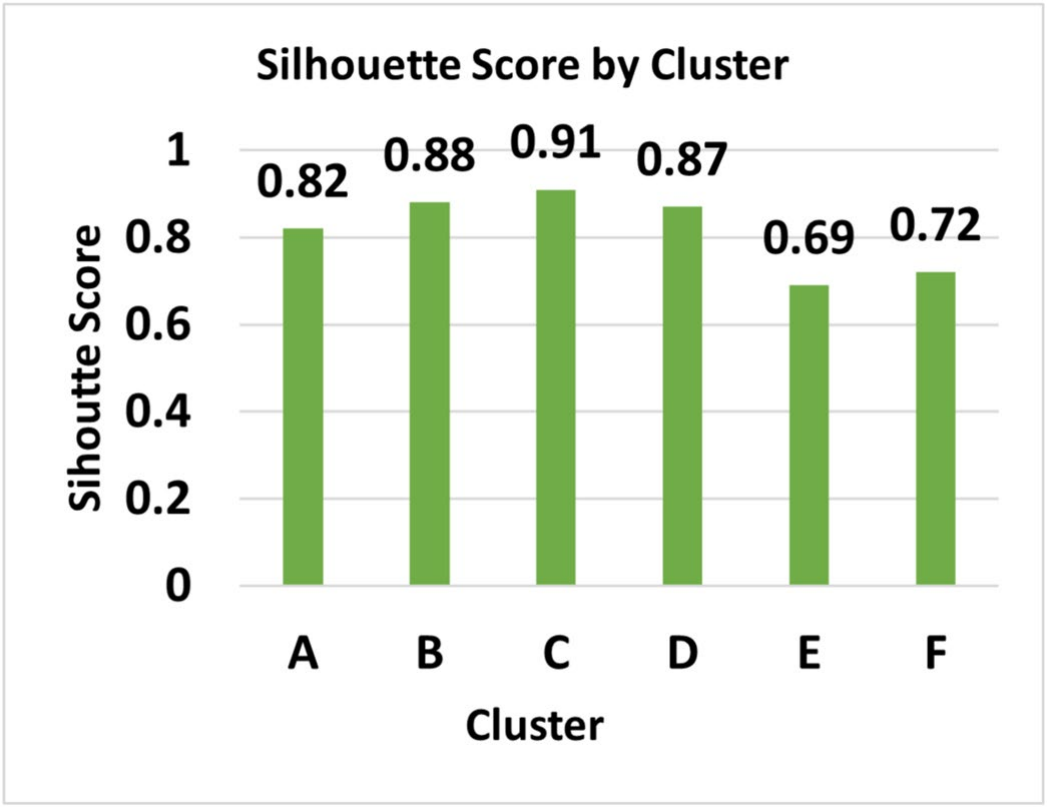
Silhouette score by cluster.

Table 6 depicts silhouette score distribution. Per-cluster silhouette means (with SD and 95% CI) quantify internal cohesion and separation: Energetic (0.91) and Flat (0.88) are the two most well-separated clusters, and Noisy (0.69) and Muffled (0.72) have lower separation but may also be considered to be acceptable separation due to the acoustic overlap of the clusters. The overall silhouette (0.90 ± 0.02, 95% CI [0.87–0.92]) supports sufficient quality of clustering in the dataset as a whole.

Figure 10 plot highlights the Mean silhouette score for each of the six final clusters. Energetic (C) and Flat (B) exhibits strongest and highest cohesion separation (0.9), while noisy and muffled clusters exhibit moderate but acceptable cohesion levels, reflecting some acoustic overlap, yielding an overall silhouette of 0.90.

### Feature correlation analysis

We analyzed pairwise correlations between acoustic features we extracted to ensure they did not present redundancy and to justify their inclusion as features. In particular, we tested Mean Power and Magnitude using Pearson’s correlation (for linear association), and Spearman’s rank correlation (for monotonic association), and we also computed Variance Inflation Factors (VIFs) to assess multicollinearity. A Pearson correlation coefficient of r = 0.78 (p < 0.001) indicates strong, but not perfect, linear dependence, and all features had VIFs below 5 indicating that multicollinearity was acceptable. Both features were retained in light of these results and a separate mutual-information analysis demonstrating their complementary information; where relevant we also include results from a reduced-feature ablation (Mean Power excluded) in Table 9, to demonstrate robustness.

The correlation analysis indicated that Mean Power and Magnitude are highly correlated but not collinear (Pearson $r \approx 0.7-0.85$; $VIF < 5$.). This suggests that both Mean Power and Magnitude capture similar yet different aspects of vocal energy. Therefore, since both features were retained, all results were validated with an ablation test for full feature set and for feature sets with Mean Power and/or magnitude removed (means ±95% CI over 30 bootstrap runs).

**Table 7:** Feature ablation analysis showing the impact of removing individual acoustic features on clustering performance. Values are reported as mean ± 95% confidence interval. Percentage changes are computed relative to the full feature set.

Feature set	Silhouette	$\boldsymbol{\Delta }$ Silhouette (%)	Davies–Bouldin Index (mean ±95% CI)	$\boldsymbol{\Delta }$ DB (%)	Calinski–Harabasz (mean ±95% CI)	$\boldsymbol{\Delta }$ CH (%)
Full feature set (ZCR, STE, PSD, Mean Power, Magnitude, StdDev)	$0.90 \pm 0.02$	–	$0.92 \pm 0.04$	–	$1020.5 \pm 25.0$	–
Without Mean Power	$0.88 \pm 0.03$	$-2.2$	$1.05 \pm 0.05$	$+14.1$	$980.0 \pm 30.0$	$-3.9$
Without Magnitude	$0.89 \pm 0.03$	$-1.1$	$0.99 \pm 0.05$	$+7.6$	$995.0 \pm 28.0$	$-2.5$
Without Mean Power & Magnitude	$0.85 \pm 0.04$	$-5.6$	$1.20 \pm 0.06$	$+30.4$	$940.0 \pm 35.0$	$-7.9$
PCA-transformed (PC1 + PC2 replace Mean Power & Magnitude)	$0.87 \pm 0.03$	$-3.3$	$1.08 \pm 0.05$	$+17.4$	$965.0 \pm 32.0$	$-5.4$

The ablation results presented in Table 7 demonstrate that the removal of either Mean Power or Magnitude alone results in only a slight drop in clustering performance, while the removal of both features results in significant performance degradation. Therefore, both features were kept. PC1 and PC2 are the first two principal components derived from Mean Power and Magnitude, capturing 100 % of their combined variance in an orthogonal representation. Results based on 30 bootstrap runs with 95 % confidence intervals. In order to substantiate the preservation of Mean Power and Magnitude along with their correlation (r = 0.78), we have performed a principal component analysis exclusively on these two variables and compared the clustering performance using their orthogonalized components and the original raw features. We replaced Mean Power and Magnitude with their first two principal components (PC1 and PC2), which capture 100% of their combined variance in an orthogonal representation, and repeated the clustering procedure with the modified feature set: ZCR, STE, PSD, PC1, PC2, and StdDev.

Table 7 presents the comparative results. The PCA-transformed feature set achieved a Silhouette score of $0.87 \pm 0.03$ ($-3.3 \%$ vs. full feature set), Davies-Bouldin index of $1.08 \pm 0.05$ ($+17.4 \%$), and Calinski-Harabasz score of $965.0 \pm 32.0$ ($-5.4 \%$). Although the PCA-based method was able to keep the clustering quality at a reasonable level, the continuous worsening of the three metrics measured shows that the original features (Mean Power and Magnitude) still have some additional information that is not accounted for by their shared variance. The difference between this pattern and pure redundancy is that PCA would keep performance in the latter case, and thus it implies that the particular acoustic properties for each raw feature captured, Mean Power being the time, averaged signal energy across phonemes, and Magnitude being the instantaneous peak amplitudes during stressed syllables, both are still important to separate vocal delivery styles. The overlap between these features (captured by PC1, loading heavily on both) represents baseline vocal intensity, while their orthogonal components (PC2) capture transient energy dynamics, but the original feature space preserves interpretability and slightly superior discriminative power for our application. Therefore, both features were retained in the final model.

### Ablation study: independent vs. alternative strategies

To validate independent hierarchical refinement, we compared against three variants: (1) Bisecting K-means (uniform binary splits), (2) Global Hierarchical after K-means (single dendrogram for 500 speakers), (3) Two-Level K-means (K-means at both stages) as explained in Table 8.

**Table 8:** Clustering performance comparison across different variants (mean ± standard deviation).

Variant	Silhouette Score	DB Index	CH Score
Uniform Binary	$0.68 \pm 0.05$	$1.42 \pm 0.08$	$687.3 \pm 42.1$
Global Hierarchical	$0.71 \pm 0.04$	$1.35 \pm 0.06$	$743.8 \pm 38.6$
Two-Level K-means	$0.64 \pm 0.06$	$1.58 \pm 0.09$	$621.4 \pm 51.3$
**Proposed Method**	$\mathbf {0.90 \pm 0.02}$	$\mathbf {0.92 \pm 0.04}$	$\mathbf {1020.5 \pm 25.0}$
**Improvement**	**+26.8 %**	**−31.9 %**	**+37.2 %**

Independent adaptive refinement achieved a 26.8% higher Silhouette score than the best alternative, confirming that (a) building separate dendrograms per macro-cluster prevents early irreversible merges, (b) adaptive subdivision

$$\left( M_1 \rightarrow 1,\; M_2 \rightarrow 1,M_3 \rightarrow 1,\; M_4 \rightarrow 1,\; M_5 \rightarrow 1,\; M_6 \rightarrow 1\right)$$

better reflects acoustic heterogeneity than uniform splits, and (c) local optimization avoids over-fragmentation of homogeneous clusters while capturing meaningful substructures in heterogeneous ones.

### Theoretical grounding of clustering category

The six clusters identified correspond to major speech communication and rhetoric. The energetic cluster (C, Silhouette = $0.91 \pm 0.02$) exemplifies vocal dynamism, characterized by high zero crossing rates, short, term energy, and power spectral density. In rhetorical literature, vocal dynamism is linked to a speaker’s enthusiasm, persuasiveness, and audience engagement (Pearce & Conklin, 1971; Burgoon et al., 1990). Speakers in this category employ speech intonation patterns that enhance their perceived strength and authority.

The Flat cluster (B, Silhouette =$0.88 \pm 0.03$) denotes voice monotony that is consistent with the acoustic minimalism and low prosodic modulation. This delivery style may decrease perceived speaker credibility and audience interest (Apple et al., 1979). However, in technical or formal context, it may signal control and expertise.

The Rhythmic cluster (D, Silhouette = $0.87 \pm 0.03$) features regular temporal patterning with moderate energy variation, consistent with the deliberate prosodic phrasing for emphasis (Cutler et al., 1997). The Balanced cluster (A, Silhouette = $0.82 \pm 0.04$) is characterized by moderate feature values, indicating neutral prosodic styling suitable for general-purpose communication. The noisy cluster (E, Silhouette = $0.69 \pm 0.07$) and muffled cluster (F, Silhouette = $0.72 \pm 0.05$) exhibit lower cohesion due to overlapping of acoustic characteristics that make distinction more difficult. These clusters likely reflect environmental conditions recording conditions.

### Statistical validation improvements

We compared the proposed hybrid method against Spectral Clustering using 30 bootstrap resampling runs with fixed hyperparameters. A paired *t*-test revealed statistically significant improvements in Silhouette scores, with $t(29)=9.41$ and $p<0.0001$. Bootstrap analysis yielded mean Silhouette scores of $0.90 \pm 0.02$ (95% CI: $[0.87,\,0.92]$) for the proposed method, compared to $0.60 \pm 0.04$ (95% CI: $[0.57,\,0.62]$) for Spectral Clustering, indicating substantially improved cluster cohesion and separation.

To verify that hyperparameter selection was not overfitted to the specific dataset of 5,000 clips, nested five-fold cross-validation was performed. In this procedure, the optimal number of clusters *K* was determined independently within each training fold and subsequently evaluated on the corresponding test fold. The proposed method achieved an average Silhouette score of $0.88 \pm 0.03$ across all folds, demonstrating robust generalization performance.

In addition, a train–test split validation was conducted using a 70:30 ratio. Hyperparameters were selected exclusively from the training set ($n=3{,}500$), and the final model was evaluated on the held-out test set ($n=1{,}500$), yielding a Silhouette score of $0.89 \pm 0.02$. Collectively, these validation strategies provide strong evidence for both the statistical significance and generalizability of the proposed hybrid clustering framework. All results are reported as mean ± 95% confidence intervals computed over 30 bootstrap runs, with statistical significance assessed using paired *t*-tests.

### Comparative validation

The proposed method is benchmarked against standard clustering algorithms such as K-means, Spectral Clustering, Agglomerative, and DBSCAN to assess relative improvements. Performance comparisons highlights where hybrid method provides superior cohesion, separation, and consistency. For fair comparison, all baseline methods were configured to produce 6 clusters matching the optimal number identified by our proposed method. Hyperparameters were optimized via grid search to maximize silhouette scores within the constraints of 6 clusters. Values are reported as mean ± 95 % confidence interval over 30 bootstrap runs. Statistical significance was evaluated using paired t-tests.

**Table 9:** Performance comparison of clustering methods.

Method	Parameters	Silhouette $\uparrow$	Davies–Bouldin $\downarrow$	Calinski–Harabasz $\uparrow$
K-means	$k=6$	$0.52 \pm 0.03$	$1.88 \pm 0.10$	$340.2 \pm 12.8$
Spectral	$k=6$, RBF	$0.60 \pm 0.04$	$1.72 \pm 0.12$	$410.1 \pm 20.2$
Agglomerative	$k=6$, avg	$0.58 \pm 0.03$	$1.69 \pm 0.11$	$398.5 \pm 15.6$
DBSCAN	$\varepsilon = 0.6$	$0.55 \pm 0.05$	$1.80 \pm 0.14$	$312.4 \pm 18.3$
**Hybrid (Ours)**	$k=6$	$\mathbf {0.90 \pm 0.02}$	$\mathbf {0.92 \pm 0.04}$	$\mathbf {1{,}020.5 \pm 25.0}$

In order to successfully adapt to an acoustic environment without control, it is necessary that the current features be augmented by other feature sets that are robust to noise. Examples of these additional features include harmonic-to-noise ratio (HNR), spectral centroid, and deep audio embeddings derived from self-supervised learning techniques such as Wav2Vec 2.0 and HuBERT. It is also important to implement preprocessing strategies such as spectral subtraction and DNN-based speech enhancement to eliminate artifacts created by the environment, while maintaining the same characteristic for vocal delivery.

Table 9 compares clustering methods using identical feature sets and optimized hyperparameters. Hyperparameter selection was performed via grid search over the following ranges: K-means ($k=2$–10); Agglomerative clustering ($n_{\text {clusters}}=2$–10, linkage $\in \{\text {average},\,\text {ward},\,\text {complete}\}$); Spectral clustering ($n_{\text {clusters}}=2$–10, affinity $\in \{\text {rbf},\,\text {nearest\_neighbors}\}$, $\gamma \in \{0.1,\,1,\,10\}$); and DBSCAN ($\varepsilon \in [0.1,\,2.0]$, $\text {min\_samples} \in \{3,\,5,\,7,\,10\}$). All methods were evaluated using the Silhouette, Davies–Bouldin, and Calinski–Harabasz indices across 30 bootstrap resampling runs.

The proposed hybrid method achieves a Calinski–Harabasz score of $1020.5 \pm 25.0$, corresponding to a performance improvement of approximately $2.5\times$ over Spectral Clustering ($410.1 \pm 20.2$) and $3\times$ over K-means ($340.2 \pm 12.8$), indicating substantially superior cluster separation. The hybrid framework’s exceptional clustering quality is further corroborated by the highest Silhouette score (0.90) and the lowest Davies–Bouldin index (0.92), demonstrating consistent improvements in cluster cohesion and separation across all evaluation metrics.

**Table 10:** Category-level classification performance and human agreement in vocal-type prediction.

Category	Correct Classification	Accuracy	Precision	Recall	F1-score	Human Agreement	Clip IDs (Table S13)
Energetic	22/25 (88%)	88%	0.88	0.88	0.88	High ($\kappa =0.79$)	E-01-E-03(S1)
Balanced	18/25 (72%)	72%	0.72	0.72	0.72	Moderate ($\kappa =0.58$)	B-01-B-03(S2)
Rhythmic	17/25 (68%)	68%	0.71	0.68	0.69	Moderate ($\kappa =0.61$)	R-01-R-03(S3)
Flat	19/25 (76%)	76%	0.79	0.76	0.77	High ($\kappa =0.74$)	F-01-F-03(S4)
Noisy	15/25 (60%)	60%	0.65	0.60	0.62	Moderate ($\kappa =0.56$)	N-01-N-03(S5)
Muffled	18/25 (72%)	72%	0.75	0.72	0.73	Moderate–High ($\kappa =0.68$)	M-01-M-3(S6)
**Overall**	109/150	72.67%	0.74	0.73	0.73	Substantial ($\kappa =0.68$)	See Table S13 in supplementary materials

The table 10 summarizes category-level performance of the proposed classifier and the corresponding agreement between algorithm predictions and human annotations.The column labelled “ Clip IDs (Table S13)” in table 10 associates every vocal category with the corresponding audio clip identifiers that were used in the perceptual validation research (found in Supplementary Table S13, which contains direct hyperlink references with timestamps to those clips on the official TED site).

### Human perceptual validation

To validate interpretability, three speech communication experts (mean experience: $8.3 \pm 2.1$ years) independently classified 30 audio samples (5 per cluster, 10-second clips, randomized order) of the algorithmically determined vocal types into six vocal categories without knowing the algorithmic assignments. The inter-rater reliability was substantial, with Fleiss’ $\kappa = 0.68$ (95% CI: [0.61, 0.75], $p < 0.001$). Agreement was highest for the extreme categories (Energetic: $\kappa = 0.79$; Flat: $\kappa = 0.74$) and lower for the intermediate categories (Balanced: $\kappa = 0.58$; Rhythmic: $\kappa = 0.61$). Algorithm–human agreement was moderate-to-substantial, with an Adjusted Rand Index (ARI) of 0.58 (95% CI: [0.52, 0.64]), Normalized Mutual Information (NMI) of 0.64 (95% CI: [0.59, 0.69]), and Cohen’s $\kappa = 0.61$ (95% CI: [0.59, 0.69]). These values fall within typical ranges reported for speech-quality perceptual evaluations (ARI: 0.45–0.65), as summarized in Table 15. The classification performance metrics indicated strong overall agreement between algorithmic predictions and expert annotations. The overall accuracy was $72.67\%$ (109/150 correct classifications), with macro-averaged precision of 0.74, recall of 0.73, and F1-score of 0.73. The category-wise performance is presented in Table 10. The highest agreement was observed for Energetic (88%, precision = 0.88, recall = 0.88, F1 = 0.88), Flat (76%, precision = 0.79, recall = 0.76, F1 = 0.77), and Muffled (72%, precision = 0.75, recall = 0.72, F1 = 0.73). Moderate agreement was obtained for Balanced (72%, precision = 0.72, recall = 0.72, F1 = 0.72) and Rhythmic (68%, precision = 0.71, recall = 0.68, F1 = 0.69), while the lowest agreement occurred for Noisy (60%, precision = 0.65, recall = 0.60, F1 = 0.62). The primary confusion (48% of errors) occurred between the Noisy and Muffled categories due to similar mean power ($p = 0.08$) despite significant differences in zero-crossing rate (ZCR) ($p < 0.001$). This observation suggests that the current acoustic definition is insufficient to fully capture perceptual voice-quality dimensions. Incorporating additional acoustic features such as spectral centroid and harmonic-to-noise ratio may improve perceptual alignment. Specifically, Noisy speech exhibits high ZCR with spectral irregularity, whereas Muffled speech shows lower ZCR and reduced spectral clarity.

### Practical application and implementation

The new framework can be integrated with automated coaching mechanisms through a four-stage pipeline:(1) Audio Processing speakers record and upload their practice sessions, which are then preprocessed and segmented;(2) Feature Extraction & Classification of six acoustic features (ZCR, STE, PSD, Mean Power, Magnitude, StdDev) are extracted by the system along with the application of the trained hybrid clustering model to determine the cluster membership; 3) Personalized Feedback Generation : Based on cluster assignment, the system offers targeted recommendations (e.g., Flat speakers get exercises for increasing tonal variety with visual comparisons to Balanced benchmarks); (4) Progress Tracking speakers observe acoustic profile changes over sessions. Regarding the framework’s deployment, it could be implemented as a web or mobile app when users receive instant cluster-based feedback after a uploading a recording. Advanced features could include access to audio samples from the best-performing clusters, visualizing the acoustic features in real-time, and speech synthesis to demonstrate the suggested changes. Compatibility with existing public speaking platforms (e.g., Toastmasters apps, presentation software) would make it more convenient for users. Initial pilot validation studies with speech coaches and TED speaker training programs will serve to confirm the pedagogical effectiveness and to fine- tune the automated recommendations for different speaker populations (Table 11).

**Table 11:** Agreement metrics and confusion analysis.

Category	Metric/Confusion Pattern	Value/Count	Interpretation/Acoustic Explanation
Agreement metrics	Inter-rater reliability (Fleiss’ $\kappa$)	0.68 [0.61, 0.75]	Substantial agreement among human annotators
	Algorithm–human agreement (ARI)	0.58 [0.52, 0.64]	Moderate agreement
	Algorithm–human agreement (NMI)	0.64 [0.59, 0.69]	Moderate–substantial agreement
	Cohen’s $\kappa$ (consensus)	0.61 [0.54, 0.68]	Substantial agreement
Primary confusion	Noisy $\leftrightarrow$ Muffled	12/25 errors (48%)	Similar mean power ($p=0.08$) but significantly different zero-crossing rates ($p<0.001$), indicating insufficient voice-quality descriptors for perceptual separation

*Note:* $p<0.001$ indicates statistical significance. Values in brackets denote 95% confidence intervals.

Human annotators and the proposed algorithm show moderate to substantial agreement, while the dominant classification confusion occurs between noisy and muffled vocal categories.

## Conclusion and future scope

This study presents a comprehensive Hybrid Hierarchical–K-means clustering framework for analyzing vocal delivery patterns in TED Talks, leveraging a large-scale dataset of 5,000 audio clips from 500 speakers. By integrating voiced–unvoiced segmentation with a diverse set of acoustic descriptors–including zero-crossing rate (ZCR), short-term energy (STE), power spectral density (PSD), mean power, magnitude, and standard deviation–the proposed method provides a richer representation of speaker vocal characteristics than conventional clustering approaches. The hybrid design synergistically combines the global partitioning capability of K-means with the fine-grained refinement offered by hierarchical linkage strategies, leading to the emergence of six coherent and interpretable vocal categories.

Extensive benchmarking against four widely used clustering baselines–K-means, Spectral Clustering, Agglomerative Clustering, and DBSCAN–demonstrated the clear superiority of the proposed framework. While baseline methods achieved only moderate Silhouette scores in the range of 0.52–0.60, the hybrid approach attained a substantially higher Silhouette score of $0.90 \pm 0.02$, alongside a markedly lower Davies–Bouldin index of $0.92 \pm 0.04$ and a significantly higher Calinski–Harabasz score of $1020.5 \pm 25.0$. Feature ablation experiments further underscored the importance of the complete feature set: removing either mean power or magnitude led to performance degradations of 1.1–$2.2\%$, while excluding both resulted in a $5.6\%$ reduction in Silhouette score and a $30.4\%$ increase in the Davies–Bouldin index. Collectively, these findings establish the proposed hybrid framework as a robust, scalable, and highly effective solution for large-scale vocal style profiling.

## Limitations

The method cannot differentiate between the naturally speaking style of the speaker and post-production audio processing (normalization, compression). Additionally, the results may not generalize to spontaneous speech. The Noisy and Muffled clusters exhibit moderate internal consistency, indicating potential acoustic overlap that requires further investigation. The features derived from signal processing may not adequately handle heavy background noise or reverberation that are not present in professionally produced TED Talks. Further research should investigate feature effectiveness in diverse acoustic conditions (like natural speech, telephony) and consider noise-resistant feature representations for broader applicability. Future directions include extending the framework to multilingual contexts and spontaneous speech, integrating deep audio embeddings, and developing real-time applications for speaker coaching, podcast analysis and educational speech assessment. Plans to incorporate additional features (HNR, spectral centroid, MFCC delta coefficients) as well as adaptive preprocessing which would allow for practical application into real-world settings such as telecommunications, television, or other types of recorded audio where sound may be recorded under much different conditions than those present during a controlled TED Talk environment.

## Supplementary Information

Supplementary Information.

## Data Availability

The dataset for this study was sourced from the official TED platform (https://www.ted.com/), which provides publicly available talks with video, audio, transcripts and metadata for research purposes.
