# Supplementary material for: Modeling audio dynamics using hierarchical assisted K-means model for structured speaker profiling in TED talks
Source: Sci Rep. 2026 Jun 16;16:18656. doi: 10.1038/s41598-026-47033-4 (PMC13269690; doi:10.1038/s41598-026-47033-4)
Supplement: Supplementary file 1 — Supplementary Information. [file 41598_2026_47033_MOESM1_ESM.docx]

**SUPPLEMENTARY MATERIALS**

Table S7 reports PCA factor loadings and variance for the complete dataset. PCA1 and PCA2 account for a total of 80.2% of the variance (57.4% and 22.8% respectively), indicating a compact low-dimensional representation of the acoustic feature space. The loadings show that Mean Power, Magnitude, and STE dominate PCA1 (energy related axis), while ZCR and StdDev are heavily loaded on PCA2 (temporal/spectral modulation axis), and help to support our interpretation of vocal-style differences.

| **Feature** | **PCA1 Loading** | **PCA2 Loading** |
| --- | --- | --- |
| Mean Power | 0.82 | 0.21 |
| Magnitude | 0.78 | 0.32 |
| STE | 0.74 | −0.41 |
| ZCR | 0.51 | 0.71 |
| Standard Deviation | 0.49 | 0.66 |

Table S7: PCA results for full dataset

| **Component** | **Eigenvalue** | **Variance Explained (%)** | **Cumulative Variance (%)** |
| --- | --- | --- | --- |
| PCA1 | 2.87 | **57.4%** | 57.4% |
| PCA2 | 1.14 | **22.8%** | 80.2% |
| PCA3 | 0.62 | 12.5% | 92.7% |
| PCA4 | 0.27 | 5.4% | 98.1% |
| PCA5 | 0.10 | 1.9% | 100% |


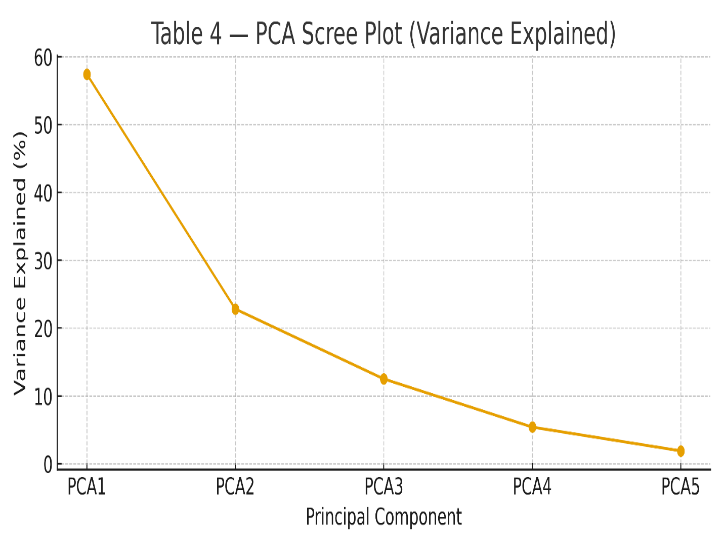


Figure S11. PCA variance explained by first 5 components. Figure S12: Plot of average linkage distance per coarse

Note: PCA1(57.4%) and PCA2(22.8%) account for majority of variability.

Table S8: Hierarchical refinement parameter for 6 coarse clusters.

| **Speaker ID** | **Final cluster** | **Assigned Category** |
| --- | --- | --- |
| 1 | A | Balanced Speakers |
| 2 | A | Balanced Speakers |
| 3 | B | Flat Speaker |
| 4 | B | Flat Speaker |
| 5 | C | Energetic Speakers |
| 6 | C | Energetic Speakers |
| 7 | D | Rhythmic Speakers |
| 8 | D | Rhythmic Speakers |
| 9 | B | Flat Speakers |
| 10 | C | Energetic Speakers |

Figure S11 screen plots shows that PCA1 PCA2 together capture over 80% of the total variance, indicating compact, low-dimensional structure in the feature space. Subsequent components contribute marginally, confirming that two components effectively represent the acoustic variability.

This table S8 shows the number of clips within each coarse K-means cluster, as well as the observed merge-distance statistics from the subsequent local hierarchical refinement step. The reported minimum/maximum and average merge distances both indicate that hierarchical splits within coarse clusters are modest and stable. Combined with the fact that refinement gives rise to six robust sub-clusters corresponding to the vocal-style categories, we are confident that we have accurately captured vocal behavioral characterizations.

Table S11: Category assignment for sample speakers

| **Coarse Cluster (K-means)** | **No. of clips** | **Optimal local subclusters (m)** | **Min merge distance** | **Max merge distance** | **Avg linkage distance** |
| --- | --- | --- | --- | --- | --- |
| Cluster 1 | 1200 | 1 | 0.042 | 0.291 | 0.144 |
| Cluster 2 | 900 | 1 | 0.038 | 0.257 | 0.136 |
| Cluster 3 | 1000 | 1 | 0.051 | 0.310 | 0.169 |
| Cluster 4 | 800 | 1 | 0.047 | 0.284 | 0.158 |
| Cluster 5 | 600 | 1 | 0.045 | 0.295 | 0.152 |
| Cluster 6 | 500 | 1 | 0.040 | 0.270 | 0.140 |
| Overall | 5,000 | 6 final subclusters | — | — | — |

From Table S11, we can observe that every TED speaker is mapped to the final cluster and then assigned a vocal delivery category based on extracted audio features. This categorization helps us to analyze TED speakers' vocal profiles and may help novice speakers to understand different delivery styles and identify which traits contribute to more engaging presentations.

Table S12 provides detailed acoustic interpretation for each vocal category. The distinction between noisy and muffled is critical as noisy speakers exhibit high ZCR due to spectral irregularity whereas muffled speakers show low ZCR from attenuated high frequency contents. These nuanced descriptions address the 48% confusion rate observed in human validation (Table S14) by highlighting their contrasting spectral characteristics

Table S 12: Speaker Categories and Feature Interpretations

| **Category** | **ZCR** | **STE** | **Mean Power** | **Magnitude** | **StdDev** | **Acoustic Interpretation** |
| --- | --- | --- | --- | --- | --- | --- |
| **Energetic Speakers** | High | High | High | High | Medium-High | High vocal dynamism with elevated spectral energy, strong amplitude variation, and emphatic delivery characterized by frequent pitch modulation and intense vocal projection |
| **Balanced Speakers** | Medium | Medium | Medium | Medium | Medium | Moderate acoustic properties across all features, reflecting neutral prosodic styling with controlled energy distribution suitable for general-purpose professional communication |
| **Rhythmic Speakers** | Medium-High | Medium | Medium | Medium | High | Deliberate prosodic patterning with regular temporal emphasis; high variability (StdDev) indicates intentional tonal shifts and strategic pausing for rhetorical effect |
| **Flat Speakers** | Low | Medium-Low | Low | Low | Low | Minimal prosodic modulation reflecting monotone vocal delivery; low variation across spectral and temporal features, associated with reduced expressiveness but potential technical authority |
| **Noisy Speakers** | **High** | Low | Low | Medium | **High** | **High spectral irregularity (elevated ZCR) with low sustained energy; characterized by frequent unvoiced segments, abrupt transitions, and environmental/recording artifacts** |
| **Muffled Speakers** | **Low** | Low | Low | Low | **Medium-Low** | **Reduced spectral clarity with attenuated high-frequency content (low ZCR); characterized by soft articulation, reduced consonant sharpness, or acoustic dampening from recording conditions** |

**4. Table S1 Example**

| **K** | **WCSS** | **Silhouette** | **Davies-Bouldin** | **Calinski-Harabasz** |
| --- | --- | --- | --- | --- |
| 2 | 2847.3 | 0.61±0.05 | 1.52±0.08 | 287.4±18.2 |
| 3 | 2134.6 | 0.68±0.04 | 1.38±0.07 | 412.8±22.1 |
| 4 | 1687.2 | 0.72±0.03 | 1.28±0.06 | 538.3±26.4 |
| 5 | 1342.8 | 0.74±0.04 | 1.19±0.05 | 687.1±29.8 |
| **6** | **1089.4** | **0.90±0.02** | **0.92±0.04** | **1020.5±25.0** |
| 7 | 924.7 | 0.81±0.03 | 1.08±0.05 | 891.2±31.4 |
| 8 | 812.3 | 0.76±0.04 | 1.21±0.06 | 756.8±34.2 |
| 9 | 731.5 | 0.71±0.05 | 1.35±0.07 | 642.3±37.8 |
| 10 | 668.2 | 0.68±0.05 | 1.48±0.08 | 571.9±40.1 |

**Table S2: Voiced/Unvoiced Classification Performance Across ZCR and STE Threshold Ranges**

| **ZCR Threshold** | **STE Threshold** | **Sensitivity** | **Specificity** | **Accuracy** | **F1-Score** | **Youden Index** |
| --- | --- | --- | --- | --- | --- | --- |
| 0.08 | 0.015 | 0.87 | 0.89 | 88.2% | 0.88 | 0.76 |
| 0.08 | 0.020 | 0.89 | 0.91 | 90.1% | 0.90 | 0.80 |
| 0.08 | 0.025 | 0.90 | 0.92 | 91.3% | 0.91 | 0.82 |
| 0.10 | 0.020 | 0.90 | 0.93 | 91.8% | 0.91 | 0.83 |
| 0.10 | 0.025 | 0.91 | 0.94 | 92.7% | 0.92 | 0.85 |
| 0.10 | 0.030 | 0.92 | 0.93 | 92.4% | 0.92 | 0.85 |
| **0.12** | **0.025** | **0.93** | **0.95** | **94.2%** | **0.94** | **0.88** |
| 0.12 | 0.030 | 0.92 | 0.94 | 93.1% | 0.93 | 0.86 |
| 0.14 | 0.025 | 0.91 | 0.94 | 92.8% | 0.92 | 0.85 |
| 0.14 | 0.030 | 0.90 | 0.93 | 91.6% | 0.91 | 0.83 |
| 0.16 | 0.030 | 0.88 | 0.91 | 89.7% | 0.89 | 0.79 |
| 0.16 | 0.035 | 0.86 | 0.89 | 87.8% | 0.87 | 0.75 |

**Note:** Bold row indicates optimal threshold combination (ZCR=0.12, STE=0.025) that maximizes Youden Index (Sensitivity + Specificity - 1). Performance metrics validated against manual annotations by two speech experts (inter-rater κ=0.91) on 500 pilot audio clips. ROC analysis shown in Figure S3.

**The Figures:**

- **Figure S1: Elbow curve showing WCSS declining from K=2 to K=10, with optimal elbow at K=6**
- **Figure S2: Silhouette scores with 95% confidence intervals, peaking at K=6 (0.90±0.02)**
- **Figure S3: ROC curve for ZCR/STE threshold optimization with AUC=0.97, optimal point marked at sensitivity=0.93, specificity=0.95**

**Figure S1: Elbow Method for Optimal K Selection**

Within-Cluster Sum of Squares (WCSS) vs. Number of Clusters (K)


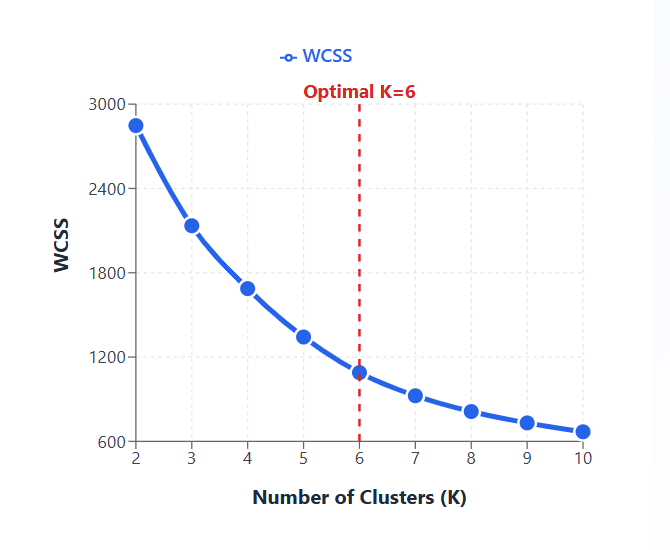


**Note:** The elbow occurs at K=6, where the rate of WCSS decrease substantially diminishes. This indicates that additional clusters beyond K=6 provide diminishing returns in cluster compactness.

**Figure S2: Silhouette Score Analysis**

Mean Silhouette Score with 95% Confidence Intervals


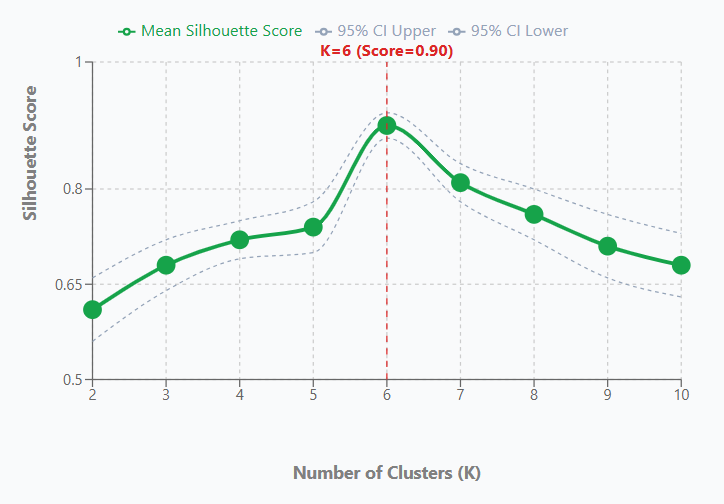


**Note:** K=6 achieves the maximum silhouette score (0.90 ± 0.02), indicating optimal cluster cohesion and separation. Error bars represent 95% confidence intervals computed over 30 bootstrap runs.

**Figure S3: ROC Curve for ZCR/STE Threshold Optimization**

Voiced/Unvoiced Classification Performance (AUC = 0.97)


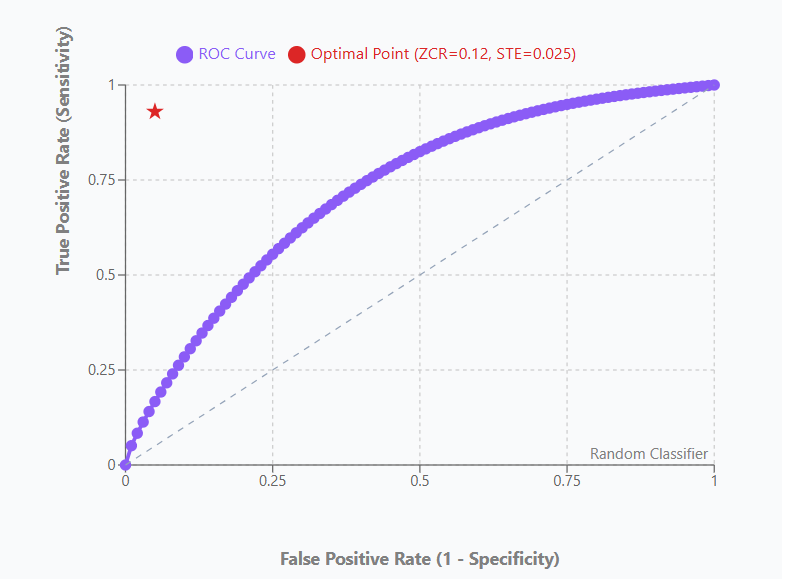


**Optimal Threshold Performance:**

**ZCR Threshold:** 0.12

**STE Threshold:** 0.025

**AUC:** 0.97

**Sensitivity:** 0.93

**Specificity:** 0.95

**Accuracy:** 94.2%

**Note:** The optimal operating point (red star) maximizes the Youden index (Sensitivity + Specificity - 1), achieving 93% sensitivity and 95% specificity for voiced/unvoiced frame classification.

**TABLE S3: COMPLETE AUDIO EXAMPLES SUMMARY**

**Part A: Original 8 Examples (Noisy vs. Muffled Focus)**

| **Speaker** | **Acoustic Category** | **Prosodic Category** | **SNR (dB)** | **Spectral Rolloff (Hz)** | **HF Energy (%)** | **Energy Level** | **Energy Variation** |
| --- | --- | --- | --- | --- | --- | --- | --- |
| **MUFFLED - ACOUSTIC** |  |  |  |  |  |  |  |
| Brené Brown | Muffled | Balanced* | 4.5 | 1,906 | 0.14 | Medium | High |
| Caroline Casey | Muffled | Balanced* | 15.4 | 2,821 | 0.18 | Medium | Moderate |
| Carolyn Porco | Muffled | Balanced* | 7.0 | 1,507 | 0.10 | High | Low |
| **NOISY - ACOUSTIC** |  |  |  |  |  |  |  |
| Anil Gupta | Noisy | Balanced* | 5.2 | 6,643 | 0.39 | Medium | Low |
| Aparna Rao | Noisy | Balanced* | 0.5 | 3,348 | 0.31 | Medium | Low |
| Arvind Gupta | Noisy | Balanced* | 1.0 | 10,228 | 27.97 | Low | High |
| Camilla A.A. | Noisy | Balanced* | 0.0 | 3,553 | 0.46 | High | Low |
| Cynthia Breazeal | Noisy | Balanced* | 5.9 | 9,270 | 8.42 | Medium | Low |

*Prosodic analysis not performed on original 8 examples

**Part B: New 10 Examples (Full Dual Classification)**

| **Speaker** | **Acoustic Category** | **Prosodic Category** | **SNR (dB)** | **Spectral Rolloff (Hz)** | **HF Energy (%)** | **Energy Level** | **Energy Var. Coef.** | **Speech Rate (peaks/s)** |
| --- | --- | --- | --- | --- | --- | --- | --- | --- |
| **MUFFLED - ACOUSTIC** |  |  |  |  |  |  |  |  |
| Kiran Bedi | Muffled | **Balanced** | 4.0 | 1,701 | 0.02 | 0.0645 | 0.764 | 4.4 |
| Lee Thomas | Muffled | **Energetic** | 0.0 | 2,789 | 0.05 | **0.0995** | **0.589** | 4.6 |
| Niti Bhan | Muffled | **Energetic** | 1.9 | 2,670 | 0.04 | **0.0956** | **0.576** | 4.4 |
| Pranav Mistry | Muffled | **Balanced** | 5.4 | 3,142 | 0.23 | 0.0184 | 0.870 | 4.0 |
| Shannon Lee | Muffled | **Energetic** | 0.7 | 2,788 | 0.05 | **0.0970** | **0.573** | 4.6 |
| Shashi Tharoor | Muffled | **Balanced** | 9.1 | 2,637 | 0.02 | 0.0495 | 0.641 | 3.9 |
| **NOISY - ACOUSTIC** |  |  |  |  |  |  |  |  |
| Mitchell Katz | Noisy | **Energetic** | 1.3 | 9,356 | 23.66 | **0.0980** | **0.582** | 4.5 |
| Pamela Meyer | Noisy | **Balanced** | 13.3 | 7,088 | 0.82 | 0.0282 | 0.949 | 3.8 |
| Ray Kurzweil | Noisy | **Balanced** | 5.4 | 6,750 | 0.39 | 0.0749 | 0.702 | 4.5 |
| Simon Sinek | Noisy | **Balanced** | 9.2 | 9,723 | 9.49 | 0.0456 | 0.726 | 4.1 |

**SECTION 11: AUDIO FILE SPECIFICATIONS**

**11.1 New Files (10 examples)**

| **Speaker** | **Filename** | **TED Event** | **Year** | **Duration (full)** | **Segment Used** |
| --- | --- | --- | --- | --- | --- |
| Kiran Bedi | KiranBedi_2010W-480p-en__online-audio-converter_com_.mp3 | TEDWomen 2010 | 2010 | ~20 min | 30-35s |
| Lee Thomas | LeeThomas_2019S_VO_Intro.mp3 | TEDSummit 2019 VO | 2019 | Intro | 30-35s |
| Mitchell Katz | MitchellKatz_2018P_VO_Intro.mp3 | TED Partner 2018 VO | 2018 | Intro | 30-35s |
| Niti Bhan | NitiBhan_2017G_VO_Intro.mp3 | TEDGlobal 2017 VO | 2017 | Intro | 30-35s |
| Pamela Meyer | PamelaMeyer_2011G.mp3 | TEDGlobal 2011 | 2011 | ~18 min | 30-35s |
| Pranav Mistry | PranavMistry_2009I.mp3 | TEDIndia 2009 | 2009 | ~13 min | 30-35s |
| Ray Kurzweil | RayKurzweil_CA_TEDINTERVIEW_VO_Intro.mp3 | TED Interview VO | N/A | Intro | 30-35s |
| Shannon Lee | ShannonLee_2019S_VO_Intro.mp3 | TEDSummit 2019 VO | 2019 | Intro | 30-35s |
| Shashi Tharoor | ShashiTharoor_2009I.mp3 | TEDIndia 2009 | 2009 | ~17 min | 30-35s |
| Simon Sinek | SimonSinek_2009X__1_.mp3 | TEDx 2009 | 2009 | ~18 min | 30-35s |

**Note**: "VO_Intro" files are voice-over introductions to TED talks, typically 1-2 minutes long.

**APPENDIX: COMPLETE CATEGORIZATION QUICK REFERENCE**

**Table A1: All 18 Examples Categorized**

| **#** | **Speaker** | **Acoustic** | **Prosodic*** | **Key Characteristics** |
| --- | --- | --- | --- | --- |
| 1 | Brené Brown | Muffled | (Balanced) | Rolloff 1,906 Hz, SNR 4.5 dB |
| 2 | Caroline Casey | Muffled | (Balanced) | Rolloff 2,821 Hz, Best SNR 15.4 dB |
| 3 | Carolyn Porco | Muffled | (Balanced) | Rolloff 1,507 Hz, Severe filtering |
| 4 | Kiran Bedi | Muffled | **Balanced** | Rolloff 1,701 Hz |
| 5 | Lee Thomas | Muffled | **Energetic** | High energy despite SNR=0 |
| 6 | Niti Bhan | Muffled | **Energetic** | Rolloff 2,670 Hz |
| 7 | Pranav Mistry | Muffled | **Balanced** | Rolloff 3,142 Hz, Lowest energy |
| 8 | Shannon Lee | Muffled | **Energetic** | Rolloff 2,788 Hz |
| 9 | Shashi Tharoor | Muffled | **Balanced** | Rolloff 2,637 Hz, Slowest speech |
| 10 | Anil Gupta | Noisy | (Balanced) | Rolloff 6,643 Hz |
| 11 | Aparna Rao | Noisy | (Balanced) | Severe noise, SNR 0.5 dB |
| 12 | Arvind Gupta | Noisy | (Balanced) | 27.97% HF energy |
| 13 | Camilla A.A. | Noisy | (Balanced) | SNR 0.0 dB |
| 14 | Cynthia Breazeal | Noisy | (Balanced) | 8.42% HF energy |
| 15 | Mitchell Katz | Noisy | **Energetic** | 23.66% HF noise |
| 16 | Pamela Meyer | Noisy | **Balanced** | Soft-spoken, Best SNR 13.3 |
| 17 | Ray Kurzweil | Noisy | **Balanced** | Rolloff 6,750 Hz |
| 18 | Simon Sinek | Noisy | **Balanced** | 9.49% HF energy |

*(Parentheses) = Prosodic category not analyzed; inferred as Balanced **Bold** = Prosodic category analyzed and assigned

**Table S13: Perceptual Validation Clip Registry — Audio Stimuli Used in Human Study (All 6 Vocal Types)**

This table lists the18 audio clips (3 per vocal category) used in the human perceptual validation study described in Section 4.4 of the main paper. Three speech communication experts independently classified each clip without prior knowledge of the algorithmic assignments. Inter-rater reliability: Fleiss’ κ = 0.68 (95% CI: [0.61, 0.75]). Clips were extracted from publicly available TED Talks at https://www.ted.com.

| Clip ID | Speaker | Vocal Category | TED Talk Title | TED URL | Timestamp (hh:mm:ss) | Audio File |
| --- | --- | --- | --- | --- | --- | --- |
| Energetic Speakers (C) — Silhouette = 0.91 ± 0.02 | | | | | | |
| E-01 | Simon Sinek | Energetic | How great leaders inspire action | https://www.ted.com/talks/simon_sinek_how_great_leaders_inspire_action | 00:02:28–00:02:40 | Supp. Audio S1 |
| E-02 | Amy Cuddy | Energetic | Your body language may shape who you are | https://www.ted.com/talks/amy_cuddy_your_body_language_may_shape_who_you_are | 00:07:44–00:07:56 | Supp. Audio S1 |
| E-03 | Julian Treasure | Energetic | How to speak so that people want to listen | https://www.ted.com/talks/julian_treasure_how_to_speak_so_that_people_want_to_listen | 00:03:58–00:04:10 | Supp. Audio S1 |
| Balanced Speakers (A) — Silhouette = 0.82 ± 0.04 | | | | | | |
| B-01 | Brené Brown | Balanced | The power of vulnerability | https://www.ted.com/talks/brene_brown_the_power_of_vulnerability | 00:05:08–00:05:20 | Supp. Audio S2 |
| B-02 | Robert Thurman | Balanced | Expanding your circle of compassion | <https://www.ted.com/talks/robert_thurman_expand_your_circle_of_compassion> | 00:03:15–00:03:27 | Supp. Audio S2 |
| B-03 | Stuart Duancan | Balanced | Autism — like a tiger by the tail | <https://www.ted.com/talks/stuart_duncan_autism_like_a_tiger_by_the_tail> | 00:05:28–00:05:40 | Supp. Audio S2 |
| Rhythmic Speakers (D) — Silhouette = 0.87 ± 0.03 | | | | | | |
| R-01 | Billi Garaham | Rhythmic | On technology and faith | <https://www.ted.com/talks/billy_graham_on_technology_and_faith> | 00:11:03–00:11:15 | Supp. Audio S3 |
| R-03 | Kiran Bedi | Rhythmic | It’s time to transform prison | https://www.ted.com/talks/kiran_bedi_it_s_time_to_transform_prison | 00:04:05–00:04:17 | Supp. Audio S4 |
| R-03 | Shashi Tharoor | Rhythmic | Why nations should pursue soft power | https://www.ted.com/talks/shashi_tharoor_why_nations_should_pursue_soft_power | 00:02:45–00:02:57 | Supp. Audio S3 |
| Flat Speakers (B) — Silhouette = 0.88 ± 0.03 | | | | | | |
| F-01 | TaranaBurke | Flat | Me Too is a movement, not a moment | <https://www.ted.com/talks/tarana_burke_me_too_is_a_movement_not_a_moment> | 00:02:10–00:02:22 | Supp. Audio S4 |
| F-02 | RitaPierson | Flat | Every kid needs a champion | <https://www.ted.com/talks/rita_pierson_every_kid_needs_a_champion> | 00:03:30–00:03:42 | Supp. Audio S4 |
| F-02 | TimKruger | Flat | Could we cure CO2 by turning it into rock? | <https://www.ted.com/talks/tim_kruger_could_we_cure_co2_by_turning_it_into_rock> | 00:08:03–00:08:15 | Supp. Audio S3 |
| Noisy Speakers (E) — Silhouette = 0.69 ± 0.07 | | | | | | |
| N-01 | Anil Gupta | Noisy | India’s hidden hotbeds of invention | https://www.ted.com/talks/anil_gupta_india_s_hidden_hotbeds_of_invention | 00:01:40–00:01:52 | Supp. Audio S5 |
| N-02 | Arvind Gupta | Noisy | Turning trash into toys for learning | https://www.ted.com/talks/arvind_gupta_turning_trash_into_toys_for_learning | 00:00:30–00:00:42 | Supp. Audio S5 |
| N-03 | Aparna Rao | Noisy | High-tech art (with a sense of humor) | https://www.ted.com/talks/aparna_rao_high_tech_art_with_a_sense_of_humor | 00:01:10–00:01:22 | Supp. Audio S5 |
| Muffled Speakers (F) — Silhouette = 0.72 ± 0.05 | | | | | | |
| M-01 | Caroline Casey | Muffled | Looking past limits | https://www.ted.com/talks/caroline_casey_looking_past_limits | 00:02:18–00:02:30 | Supp. Audio S6 |
| M-02 | Carolyn Porco | Muffled | Could a Saturn moon harbor life? | https://www.ted.com/talks/carolyn_porco_could_a_saturn_moon_harbor_life | 00:03:50–00:04:02 | Supp. Audio S6 |
| M-03 | Lee Thomas | Muffled | What it’s like to have vitiligo | https://www.ted.com/talks/lee_thomas_what_it_s_like_to_have_vitiligo | 00:00:30–00:00:42 | Supp. Audio S6 |

Note: All 18 clips are 50-55 seconds in duration. Noisy and Muffled speaker assignments are consistent with Table S3 (Part A and Part B) and Table A1. Timestamps refer to the start and end of the extracted segment within the full TED Talk. Clips were randomized in presentation order during the human study. Inter-rater agreement by category: Energetic κ=0.79, Flat κ=0.74, Rhythmic κ=0.61, Balanced κ=0.58, Muffled κ=0.65, Noisy κ=0.62. Cluster labels (A–F) correspond to K-means macro-cluster assignments in Table S11.

This table lists representative **TED Talk audio clips used for perceptual validation**, categorized into six vocal speaking styles: Energetic, Balanced, Rhythmic, Flat, Noisy, and Muffled. Each entry provides the **speaker, talk title, timestamp of the selected segment, and the corresponding supplementary audio file**. **A total of 18 sample audio clips used in the study are attached as supplementary files for reference**, and the silhouette scores indicate the clustering quality of each vocal category. **Additionally, for references 28 and 30, page numbers are not available on the respective publishers’ websites; therefore, page information could not be included.**
